# The Power of the Heterogeneous Stock Rat Founder Strains in Modeling Metabolic Disease

**DOI:** 10.1210/endocr/bqad157

**Published:** 2023-10-26

**Authors:** Valerie A Wagner, Katie L Holl, Karen C Clark, John J Reho, Hans-Joachim Lehmler, Kai Wang, Justin L Grobe, Melinda R Dwinell, Hershel Raff, Anne E Kwitek

**Affiliations:** Department of Physiology, Medical College of Wisconsin, Milwaukee, WI 53226, USA; Department of Physiology, Medical College of Wisconsin, Milwaukee, WI 53226, USA; Cardiovascular Center, Medical College of Wisconsin, Milwaukee, WI 53226, USA; Department of Medicine, Medical College of Wisconsin, Milwaukee, WI 53226, USA; Department of Physiology, Medical College of Wisconsin, Milwaukee, WI 53226, USA; Comprehensive Rodent Metabolic Phenotyping Core, Medical College of Wisconsin, Milwaukee, WI 53226, USA; Department of Occupational and Environmental Health, University of Iowa, Iowa City, IA 52242, USA; Department of Biostatistics, University of Iowa, Iowa City, IA 52242, USA; Department of Physiology, Medical College of Wisconsin, Milwaukee, WI 53226, USA; Cardiovascular Center, Medical College of Wisconsin, Milwaukee, WI 53226, USA; Comprehensive Rodent Metabolic Phenotyping Core, Medical College of Wisconsin, Milwaukee, WI 53226, USA; Department of Biomedical Engineering, Medical College of Wisconsin, Milwaukee, WI 53226, USA; Department of Physiology, Medical College of Wisconsin, Milwaukee, WI 53226, USA; Cardiovascular Center, Medical College of Wisconsin, Milwaukee, WI 53226, USA; Department of Physiology, Medical College of Wisconsin, Milwaukee, WI 53226, USA; Cardiovascular Center, Medical College of Wisconsin, Milwaukee, WI 53226, USA; Department of Medicine, Medical College of Wisconsin, Milwaukee, WI 53226, USA; Endocrine Research Laboratory, Aurora St. Luke's Medical Center, Advocate Aurora Research Institute, Milwaukee, WI 53233, USA; Department of Physiology, Medical College of Wisconsin, Milwaukee, WI 53226, USA; Cardiovascular Center, Medical College of Wisconsin, Milwaukee, WI 53226, USA; Department of Biomedical Engineering, Medical College of Wisconsin, Milwaukee, WI 53226, USA; Rat Genome Database, Medical College of Wisconsin, Milwaukee, WI 53226, USA

**Keywords:** birth weight, fat distribution, BAT, thyroid hormones, RMR, genetic background

## Abstract

Metabolic diseases are a host of complex conditions, including obesity, diabetes mellitus, and metabolic syndrome. Endocrine control systems (eg, adrenals, thyroid, gonads) are causally linked to metabolic health outcomes. N/NIH Heterogeneous Stock (HS) rats are a genetically heterogeneous outbred population developed for genetic studies of complex traits. Genetic mapping studies in adult HS rats identified loci associated with cardiometabolic risks, such as glucose intolerance, insulin resistance, and increased body mass index. This study determined underappreciated metabolic health traits and the associated endocrine glands within available substrains of the HS rat founders. We hypothesize that the genetic diversity of the HS rat founder strains causes a range of endocrine health conditions contributing to the diversity of cardiometabolic disease risks. ACI/EurMcwi, BN/NHsdMcwi, BUF/MnaMcwi, F344/StmMcwi, M520/NRrrcMcwi, and WKY/NCrl rats of both sexes were studied from birth until 13 weeks of age. Birth weight was recorded, body weight was measured weekly, metabolic characteristics were assessed, and blood and tissues were collected. Our data show wide variation in endocrine traits and metabolic health states in ACI, BN, BUF, F344, M520, and WKY rat strains. This is the first report to compare birth weight, resting metabolic rate, endocrine gland weight, hypothalamic–pituitary–thyroid axis hormones, and brown adipose tissue weight in these rat strains. Importantly, this work unveils new potential for the HS rat population to model early life adversity and adrenal and thyroid pathophysiology. The HS population likely inherited risk alleles for these strain-specific traits, making the HS rat a powerful model to investigate interventions on endocrine and metabolic health.

Obesity, defined in humans as a body mass index (BMI) ≥ 30.0 kg/m^2^, is a complex genetic syndrome the etiology of which interacts with environmental factors. The prevalence of self-reported obesity in 2021 among US adults ranged from 24.7% in the District of Columbia to 40.3% in Kentucky (1). The prevalence of obesity in children and adolescents aged 2-19 years in the United States from 2017 to March 2020 was 19.7% (2). Moreover, increased BMI has been implicated in 4.7 million deaths worldwide in 2017 (3), making obesity a severe threat to human health. The World Obesity Atlas estimates that obesity will increase from 14% to 24% of the global population by 2035, affecting approximately 2 billion children, adolescents, and adults (4). Inheritance patterns account for 40% to 70% of BMI variation in humans (5, 6) and regulates interacting neuroendocrine systems and metabolic pathways evolved to store energy from food. These genetic factors interact with overnutrition, sedentary behavior, and environmental factors that disrupt energy balance (7), leading to the obesity epidemic.

At its core, obesity is endocrine system dysfunction as adipose tissue receives input from hormones from the adrenals and thyroid (8). Perturbations in pathways regulating energy balance and the presence of comorbidities could independently disrupt homeostasis and result in obesity as a common outcome (9, 10). Evaluation of these complex disease states are challenging in the human population due to significant interactions of multiple body systems, uncontrolled environmental and therapeutic factors, and high genetic diversity. In contrast, common laboratory animal models typically lack the genetic diversity required to translate findings to human populations. Thus, incorporating genetic variation in metabolic health studies is crucial to advance precision health initiatives.

Genetically tractable, population-based animal models make studying complex traits feasible in the laboratory setting and offer considerable advantages in modeling heterogeneous diseases with complex genetic mechanisms (11). The NIH created the Heterogeneous Stock (HS) rats as a population-based model for experimental and selection studies by systematically outcrossing 8 inbred rat strains to maintain genetic variation (12). The HS founder strains (ACI/N, BN/SsN, BUF/N, F344/N, M520/N, MR/N, WKY/N, WN/N) were chosen based on their phenotypic differences, and the genetic differences between the founder strains encompass much of the rat phylogenetic tree (13). Each HS rat represents a genetic mosaic of the 8 founders, with genetic diversity more closely representing humans than other available rat models (14). Mapping studies using the HS rat have identified potential candidate genes for complex traits like body weight, BMI, adiposity, fasting glucose, glucose tolerance, insulin resistance, leptin, and the response to high-fat diet challenge (15‐20). Previous work also demonstrated high concordance between HS rat and human BMI-associated adipose tissue transcripts (21), suggesting excellent translatability of results from the HS rat to human populations.

The HS founder strains possess a wide variety of known disease risks beyond obesity-related traits. ACI (August Copenhagen Irish) rats show an 11% incidence of renal agenesis, a developmental malformation of the urogenital tract (22, 23), and are a popular model for allograft and transplantation biology (24, 25). The BN (Brown Norway) strain exhibits congenital hydronephrosis and reproductive dysfunction, with male BN rats developing primary and secondary testicular failure and low circulating testosterone in adulthood (26‐29). The BUF (Buffalo) strain is known to develop spontaneous autoimmune thyroiditis, hyperplasia of the thymus and peripheral T-cell system, spontaneous thymoma, and focal and segmental glomerulosclerosis (30‐34). F344 (Fisher 344) rats are an obesity model with leptin resistance and exhibit hyperreactivity to stress (35, 36). MR (Maudsley Reactive) rats are known for their high emotionality and high preference for ethanol (37, 38). M520 (Marshall 520) rats have high preference for ethanol, low blood pressure, and high incidence of renal disease (23, 38, 39). WKY (Wistar/Kyoto) rats have significant hypothalamic–pituitary–axis dysfunction and depression-like behaviors (40‐42). WN (Inbred Wistar) rats have high incidence of pituitary tumors in females and of severe chronic nephritis (43, 44).

Unfortunately, the health profiles of the HS founder strains often lack hormone assessment levels beyond adipokines, endocrine gland weights, metabolic rate, and brown adipose tissue weight among other metabolic phenotypes. These understudied features of metabolic and endocrine health are key to understanding the full potential of the HS rat to model metabolic disease. Available phenotyping data in the HS founders often excludes females, which is a critical gap in knowledge as metabolic disease in females can present differently than in males (45). Evidence from the Collaborative Cross mice, another population model, indicates that genetic mechanisms of obesity can be sex specific (46), prompting further inclusion of females in metabolic health studies. While the original N substrains of the HS rat founders no longer exist, there are substrains with high genetic identity with the founders that likely have similar health traits (47). In this study, we determined novel metabolic and endocrine health characteristics in both sexes of 6 available substrains similar to the HS rat founders. This deep-phenotyping protocol provides new insight into the exceptional potential of the HS rat population to model complex metabolic health states. We hypothesize that the genetic diversity in the HS rat founder strains represents a range of endocrine health conditions contributing to the diversity of cardiometabolic disease risks exhibited in the HS rat population.

## Materials and Methods

### Animals

This is the second of a series of studies in a vehicle–control cohort from a previously published chemical exposure study in Wagner et al (48). In this presentation, all phenotyping data, tissue samples, and blood samples from animals 3 weeks of age and older are from animals in Wagner et al (48) but underwent new analyses to compare across strains. Hormone level and reverse transcription quantitative polymerase chain reaction (RT-qPCR) experiments presented here are new experiments not performed in Wagner et al (48). Breeding colony and birth weight data were collected independently of Wagner et al (48).

ACI/EurMcwi (ACI; RRID:RRRC_00284), BN/NHsdMcwi (BN; RRID:RGD_61498), BUF/MnaMcwi (BUF; RRID:RGD_156403001), F344/StmMcwi (F344; RRID:RGD_39456105), and M520/NRrrcMcwi (M520; RRID:RGD_40818401) male and female rats were purchased from the Hybrid Rat Diversity Program at the Medical College of Wisconsin between 3 to 4 weeks of age. WKY/NCrl (WKY; RRID:RGD_1358112) male and female rats were purchased from Charles River Laboratories between 3 and 4 weeks of age. The MR strain has not yet been cryo-resuscitated, and the WN strain is extinct, so these 2 strains were not studied. All rats used for breeding were maintained on ad libitum chlorinated reverse-osmosis water and phytoestrogen-free Teklad 2920X diet (ENVIGO, Indianapolis, IN; <20 mg/kg isoflavones). Breeding pairs were set up between 8 and 13 weeks of age. Offspring rats were weaned at 3 weeks of age, singly housed, and provided Teklad 2920X and 0.1% ethanol drinking water in glass water bottles (vehicle conditions from Wagner et al (48)). Animals were housed in polysulfone micro-isolater cages with wood chip bedding on a 14:10 light/dark cycle and were provided paper huts (Bio-Huts for Rats, Certified, Bio-Serv, Flemington, NJ), paper packets (ENVIROPAK, W.F. Fisher and Son, Branchburg, NJ), and nylon bones (K3581, Bio-Serv, Flemington, NJ) as environmental enrichment. Final sample sizes were 9 to 17 females and 10 to 15 males per strain. ACI rats display renal agenesis in ∼11% of the strain, a congenital disorder that involves abnormal development of the urogenital tract (22, 23). ACI rats with renal agenesis were removed from the study (3 ACI males and 2 ACI females). For the birth weight analysis, birth weights from pups born to ACI dams with renal agenesis were removed from the dataset. All experiments were conducted in accordance with the Guide for the Care and Use of Laboratory Animals in a protocol approved by the Institutional Animal Care and Use Committee at the Medical College of Wisconsin (49).

### Phenotyping Protocol

Birth weights were assessed within 24 hours of birth, at which time the pups were sexed based on anogenital distance (AGD). Body weight was measured weekly beginning at 1 week of age. Body composition was assessed at 3 (weaning), 4, 10, and 11 weeks of age using time domain nuclear magnetic resonance (TD-NMR; LF110, Bruker Biospin, Billerica, MA). Note that 3- and 4-week-old animals were measured using the TD-NMR mouse probe (calibrated total body fat range: 1.576-66.81 g), and the 10- and 11-week-old animals were measured using the TD-NMR rat probe (calibrated total body fat range: 3.082-336 g). After NMR, animals were placed in metabolic cages (#40615, Lab Products, Inc.) at 4 and 10 weeks of age and provided 0.1% ethanol drinking water and food (Teklad 2920X, ad libitum). After a 48-hour acclimation period, 24-hour urine and fecal samples were collected and weighed. Fecal samples were stored at −20 °C along with a representative sample of diet. Neat urine samples were centrifuged at 4000*g* for 10 minutes, aliquoted, and stored at −80 °C. Food and drinking water consumed in the 24-hour period was determined. Feeding and drinking behaviors, energy expenditure, and ambulation were measured using a multiplexed metabolic phenotyping system (Promethion, Sable Systems International, Las Vegas, NV) at 11 weeks of age (described below). After an overnight fast at 13 weeks of age, blood was obtained from the saphenous vein in unanesthetized animals and blood glucose was measured using a handheld glucometer (Contour Blood Glucose Meter, Bayer, Leverkusen, Germany). Animals were then anesthetized with CO_2_ and a thoracotomy performed. Blood samples were collected by cardiac puncture between 07:00 and 14:00, processed into serum (BD Microtainer, SST-Amber, Franklin Lakes, NJ) or plasma (BD Microtainer, Tubes with K2E (K_2_EDTA), Franklin Lakes, NJ), aliquoted, and stored at −80 °C. Note that the average blood collection time was 09:30 to 10:30 for each sex and strain group. Body length measures (nose to rump, nose to tip of tail, tibia) were determined. Tissues (liver, left ventricle of the heart, kidneys, gonads, adrenal glands, gonadal white adipose tissue [GWAT], perirenal white adipose tissue [PWAT], thymus gland, thyroid gland, hypothalamus, pituitary gland, soleus muscle, inguinal white adipose tissue [IWAT], and interscapular brown adipose tissue [BAT]) were harvested and weights collected on liver, left ventricle of the heart, kidneys, gonads, adrenal glands, GWAT, PWAT, thymus gland, thyroid gland, pituitary gland, IWAT, and BAT. Tissue samples were either placed in RNAlater (Invitrogen, Carlsbad, CA) or snap frozen in liquid nitrogen, and all were stored at −80 °C for RT-qPCR.

### Calorimetry

To quantitatively assess digestive efficiency and caloric absorption, bomb calorimetry was performed on fecal samples at the Comprehensive Rodent Metabolic Phenotyping Core at the Medical College of Wisconsin as previously described (50, 51). Briefly, 24-hour fecal samples collected in metabolic cages were desiccated in an oven and weighed prior to and after desiccation to determine water content. Desiccated samples were pressed into pellets and weighed. Digestive efficiency and total daily caloric absorption were determined using a semi-micro bomb calorimeter (Model 6725, Parr Instrument Co., Moline, IL) by combusting samples to completion according to the manufacturer's protocol. Desiccated, powdered Teklad 2920X diet samples obtained at the time of metabolic cages were also analyzed by bomb calorimetry to determine total caloric density of the specific lot of diet used in the study.

### Promethion Multiplexed Metabolic Phenotyping

Subsets of animals were phenotyped at 11 weeks of age using a multiplexed metabolic phenotyping system (Promethion, Sable Systems International, Las Vegas, NV) at the Comprehensive Rodent Metabolic Phenotyping Core at the Medical College of Wisconsin. Animals were provided ad libitum Teklad 2920X and 0.1% ethanol drinking water while in the Promethion housing system. On Monday morning, animals entered the Promethion housing and were housed continuously until Friday morning. Body composition was recorded using TD-NMR before entering and after exiting the Promethion (51). Promethion data presented were analyzed as 24-hour measures from 6 Am Thursday to 6 Am Friday to provide maximal acclimation time of ∼72 hours. Food and fluid intake, energy expenditure, and ambulation were analyzed using custom macros supplied by Sable Systems. Meal count, food intake rate, and meal duration were extracted from the Sable Systems Food Intake Pattern Analysis. Energy expenditure was calculated using the modified Weir equation (52). Not all animals successfully acclimated to the Promethion housing; fluid intake was highly variable within the first 48 hours of housing. Animals with <10 g total fluid intake in the first 48 hours were removed from the Promethion system due to concerns over acute dehydration. Therefore, Promethion phenotyping data for the ACI and BN strains will not be presented because the acclimation success rate for these strains was 30% to 50%. Sample sizes for the remaining strains were BUF = 10, F344 = 12, M520 = 7, and WKY = 10 for the males and were BUF = 9, F344 = 6, M520 = 7, and WKY = 9 for the females. Note that 1 M520 male and 1 WKY female were removed due to dehydration concerns, reducing the M520 male sample size to 6 and the WKY female sample size to 8.

### Thyroid-Stimulating Hormone, Total Triiodothyronine, and Total Thyroxine

Rat thyroid-stimulating hormone (TSH, ng/mL) was measured by enzyme-linked immunosorbent assay in serum samples following the manufacturer's protocol (RRID:AB_2940787, Catalog #80564, Crystal Chem, Elk Grove Village, IL). This solid-phase enzyme-linked immunometric assay uses a rat anti-TSH-specific monoclonal antibody and horseradish peroxidase-labeled antibody. The sensitivity of the assay is 0.1 ng/mL, and the within-run and total precision coefficient of variations (CVs) are <10%. Total triiodothyronine (total T3, µg/dL) and total thyroxine (total T4, ng/dL) were measured by solid phase radioimmunoassay (RIA) in neat serum samples following the manufacturer's protocol (total T3 kit: 06B-256447, RRID:AB_2940789; total T4 Kit: 06B-254029, RRID:AB_2940788; MP Biomedicals, Orangeburg, NY). These assays use antibody-coated tubes and ^125^I tracer solutions. The T3 RIA sensitivity is 6.7 ng/dL and the intraassay and interassay precision CVs ranged from 4.4% to 7.5%. The T4 RIA sensitivity is 0.76 µg/dL and the intra-assay and interassay precision CVs ranged from 3.3% to 11.4%.

### RNA Isolation

BAT and IWAT tissues in RNAlater were thawed on ice. Approximately 70 mg of BAT or 150 mg of IWAT was homogenized in 1 mL or 5 mL of TRIzol Reagent (Invitrogen, Carlsbad, CA), respectively, using a Fisherbrand Bead Mill 4 Homogenizer (ThermoFisher Scientific, Waltham, MA). Homogenization was repeated, and the homogenates were cooled on ice. Samples were centrifuged at 21 100*g* for 3 minutes to pellet unhomogenized tissue. The supernatant was combined equally with chloroform (ThermoFisher Scientific, Waltham, MA), mixed rapidly by inversion, incubated at room temperature for ∼3 minutes, and centrifuged at 12 000*g* and 4 °C for 15 minutes. Total RNA was purified from the aqueous layer following the RNeasy Mini Kit (Qiagen, Hilden, Germany) according to the manufacturer's protocol. Samples were treated with DNase I from the RNase-Free DNase Set (Qiagen, Hilden, Germany) according to the manufacturer's protocol to remove gDNA. RNA was quantified by a NanoDrop 1000 Spectrophotometer (ThermoFisher Scientific, Waltham, MA) and stored at −80 °C.

### Reverse Transcription Quantitative Polymerase Chain Reaction

Purified RNA (∼1 µg) was reverse transcribed using iScript cDNA Synthesis Kit (Bio-Rad Laboratories, Hercules, CA). All RT-qPCR assays were prepared following the manufacturer protocol using Prime Time Gene Expression Master Mix gene expression assay (Integrated DNA Technologies, Coralville, Iowa). Experiments were conducted on a QuantStudio 6 Flex Real-Time PCR System (Applied Biosystems, Foster City, CA). Primer sequences for all genes are listed elsewhere (Table S1 (53)). Genes were selected to assess adaptive thermogenesis and thyroid hormone response in BAT and *Ucp1* as a white adipose beiging marker in IWAT. *Rplp0* was the endogenous control gene for both BAT and IWAT experiments. Relative mRNA expression for each gene was calculated using the comparative cycle threshold (Ct) method (54) on the average of 3 technical replicates per sample. The majority of IWAT *Ucp1* qPCR reactions resulted in “undetermined” Ct values, or nondetects. Ct values representing the average Ct for nondetect reactions were imputed using the R package nondetects v2.28.0 using probit regression (55). PCR grade water (Invitrogen, Carlsbad, CA) was used as a negative control replacing the reverse transcriptase, and rat universal RNA (Biochain, Newark, CA) was used as a positive control.

### Calculations

To normalize body weight curves, body weight gain was calculated by subtracting body weight at 1 week of age (g) from all other time points. To remove body weight as a confounding variable in tissue weights, tissue weights were normalized to body weight (g) to provide a “relative” value in units/g body weight (56). Relative AGD was calculated by normalizing birth AGD (cm) to birth weight (g). Digestive efficiency, body weight gained between 4 and 10 weeks of age, food intake between 4 and 10 weeks of age, energy efficiency, and unaccounted calories (calories expended that are not attributable to ambulation or growth) were calculated following equations in Grobe (50), Reho et al. (51), and Soto et al. (57) to assess energy balance. “Total” abdominal white adipose tissue was calculated by summing GWAT (g) and PWAT (g). Fat distribution was calculated as the ratio of total IWAT (g) to total AbWAT (g) and transformed by log_10_ (58).

### Statistical Analysis

#### Impact on experiment number

Details regarding technical outlier removal and other impacts on experimental number are shown elsewhere (Table S2 (53)).

#### Linear modeling of birth weights

Linear modeling was used to correct raw birth weight data for influencing factors (Table S3, Fig. S1 (53)). Each strain was analyzed separately using the same linear model equation:


$$y_{klp}=\;B_{lp}+X_{k}+S_{l}+e_{klp}$$

where y*_klp_* is the average birth weight of the *k*th sex of the *l*th litter of the *p*th breeder pair, B*_lp_* is the random effect of the *l*th litter of the *p*th breeder pair, S*_l_* is the size of the *l*th litter at birth, X*_k_* is the *k*th sex, and e*_klp_* is a residual term (Table S4 (53)). Model residuals were tested for normality using the Shapiro–Wilk test (α=.05) and homoscedasticity between the sexes using Leven's test (α = .05). Estimated least squares means were extracted for the sex variable. Modeling was performed in R 4.0.2 using the lme4 and lsmeans packages.

#### Linear modeling of resting metabolic rate

To correct metabolic rate measures for covariates, such as sex, fat-free mass, fat mass, 24-hour food intake, 24-hour distance traveled, 24-hour sleep time, and unaccounted calories, a generalized linear model was fit to the data. Covariates were selected after ANOVA testing of the model coefficients where F < 0.05; therefore, resting metabolic rate (RMR) was corrected for fat-free mass, fat mass, and 24-hour distance traveled. Estimated least squares means were extracted for the strain variable (50). Modeling was performed in R 4.0.2 using the lsmeans package.

#### Analysis of Variance

Body weight, body weight gains, body composition, Promethion data, and hormone levels were analyzed by 2-way analysis of variance (ANOVA) with Šídák's multiple comparisons test when all data were present. If the ANOVA assumption of equal variances and normality of residuals were violated or if a group was missing, data were analyzed by Kruskal–Wallis test with Dunn's multiple comparisons test in the sexes separately. RT-qPCR results were determined on the ΔCt values using 2-way ANOVA with Šídák's multiple comparisons test. Adjusted metabolic rate data were analyzed using Welch's 1-way ANOVA with Dunnett's T3 multiple comparisons test. Alpha level was .05 for all statistical testing. Analysis and data figures were created in GraphPad Prism v9.5.1.

## Results

### Body Weight and Composition Variation Begins Before Weaning

**Figure 1.**
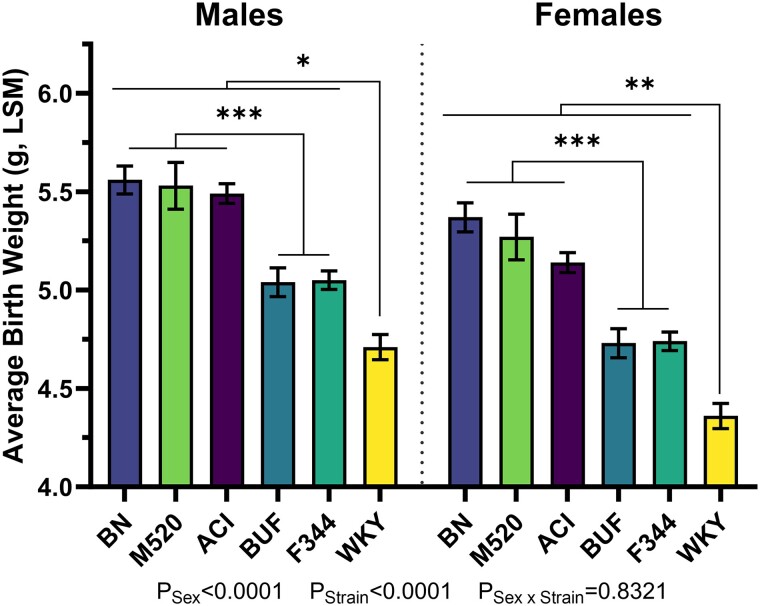
Average birth weights in male and female ACI, BN, BUF, F344, M520, and WKY rats. Body weights were taken at birth, and pups were sexed based on anogenital distance (Table S5). Least squares mean (LSM) was estimated for each strain using a linear model incorporating sex, litter size at birth, and parent identity. The estimated LSM and SEM are plotted in this figure. Birth weights in BUF, F344, and WKY rats were significantly lower than in ACI, BN, and M520 rats, with WKY rats having the lowest birth weight of all strains. Two-way ANOVA/Šídák's. n = 31-52 litters/sex/strain. *P < .05, **P < .01, ***P < .001.

Birth weights were measured from litters across all strains, and birth weights were analyzed by linear modeling within each strain including sex of the pups, litter size at birth, and parent identity as factors (Table S4 (53)). Sex at birth was determined using AGD (Table S5 (53)). Sex was a significant factor in birth weights in all strains. Breeder pair identity was a significant factor in determining birth weight in M520 and WKY litters (*P* < .01), was a trending factor in BN litter (*P* = .051), and was not significant in ACI, BUF, and F344 litters. Linear modeling analysis revealed that litter size was significant in determining birth weight with the notable exception of the BUF strain (*P* = .47, see Fig. S2 (53) for correlation plots). After correction for litter size at birth and parent identity, 3 groupings of birth weights were identified: ACI, BN, and M520 pups were similar in birth weight to each other, BUF and F344 pups were similar in birth weight but lower than ACI, BN, and M520 pups, while WKY pups were the smallest at birth compared with all other strains (all comparisons to WKY *P*_Males_ < .05, *P*_Females_ < .01, Fig. 1, Table 1).

**Table 1.** Average birth weights in male and female ACI, BN, BUF, F344, M520, and WKY rats

	Males	Females
ACI	5.49 ± 0.05 (52)	5.14 ± 0.05 (50)
BN	5.56 ± 0.07 (46)	5.37 ± 0.07 (41)
BUF	5.04 ± 0.07 (34)	4.73 ± 0.07 (33)
F344	5.05 ± 0.05 (35)	4.74 ± 0.05 (35)
M520	5.53 ± 0.12 (28)	5.27 ± 0.12 (31)
WKY	4.71 ± 0.06 (52)	4.36 ± 0.06 (51)

Birth weights were corrected for litter size at birth and parent identity for each strain using a linear model. Least squares mean estimates are presented in the following table (mean ± SEM; n = litter count).

**Figure 2.**
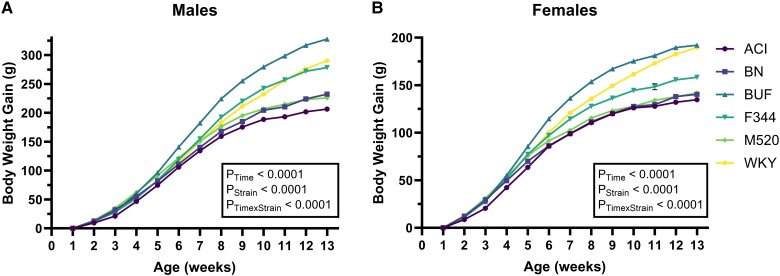
Body weight gain curves in male and female ACI, BN, BUF, F344, M520, and WKY rats. (A, B) Body weights were measured from 1 to 13 weeks of age. Body weight gain curves were constructed for male (A) and female (B) rats by correcting for body weight at 1 week of age. Significant strain differences in body weight gained over time show that BUF rats gain the most body weight and ACI males gain the least weight of the male groups. Mixed-effects model (restricted mamimum likelihood), n = 9-15/sex/strain.

Body weight gain curves were constructed by correcting for body weights taken at 1 week of age (Fig. 2, Table S6 (53, 59)). In both sexes, BUF rats attained the highest body weight over time, although WKY females reached the BUF female body weight by 13 weeks of age. WKY body weight gain curves showed the steepest slope compared with all other strains and do not appear to plateau at 13 weeks of age like both sexes in all other strains. At 13 weeks of age, F344 and WKY males had gained similar body weight. F344 females had a body weight curve that was lower than BUF and WKY, but greater than ACI, BN, and M520. BN and M520 rat body weight gain curves were similar to each other in both sexes but total body weight gain was lower than BUF, F344, and WKY rats. ACI rats tended to have the lowest body weight gain out of all strains.

**Figure 3.**
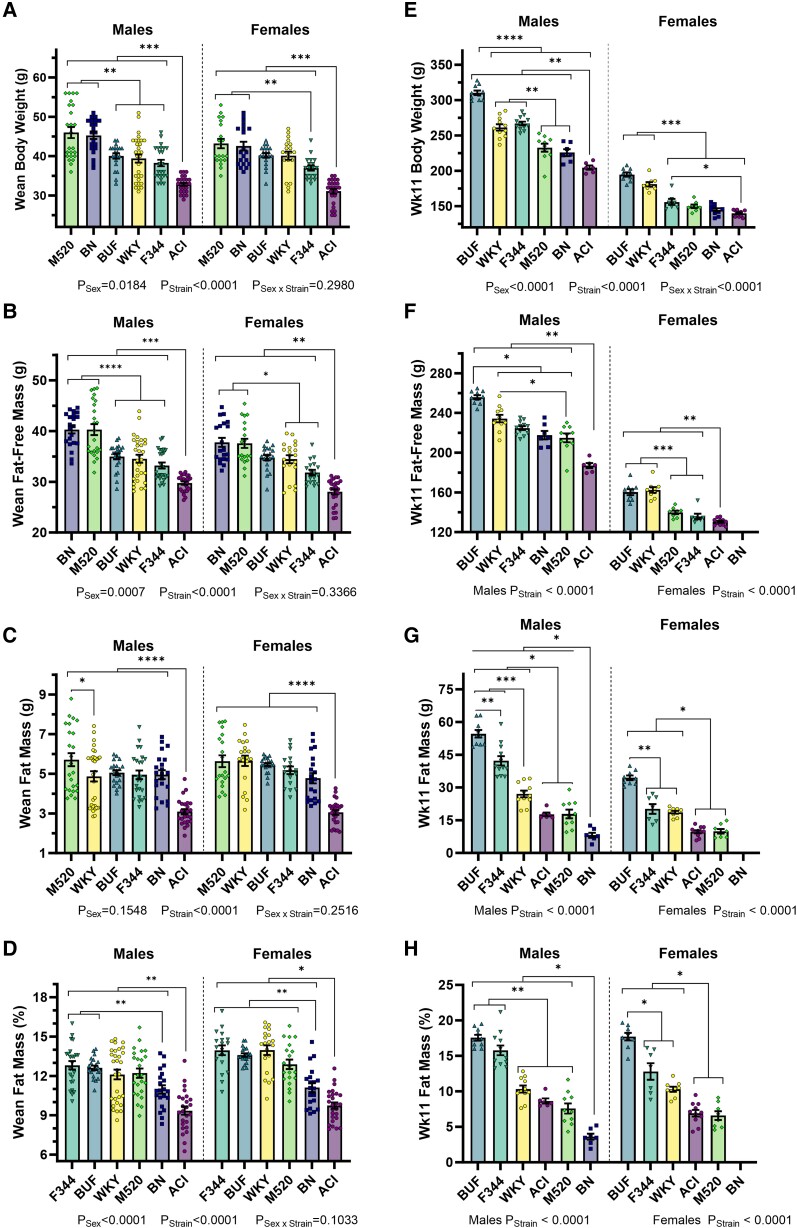
Body composition in weanling and young adult male and female ACI, BN, BUF, F344, M520, and WKY rats. Body composition was assessed by TD-NMR at 3 weeks of age (A-D) and at 11 weeks of age (E-H). Body weight (A, E), fat-free mass (B, F), fat mass (C, G), and fat mass as a percentage of body weight (D, H) are shown. At 3 weeks of age, ACI rats have lower body weight and fat mass than all other strains. At 11 weeks of age, BUF and F344 rats have the greatest fat mass, while BN rats have the least fat mass. BN females are not shown at the 11 weeks of age time point because majority of the females’ fat mass was below the calibrated range on the TD-NMR instrument. (A-E) Two-way ANOVA/Šídák's. (F-H) Welch's 1-Way ANOVA/Dunnett's by sex; 3 weeks of age n = 18-34/sex/strain, 11 weeks of age n = 3–12/sex/strain. *P < .05, **P < .01, ***P < .001, ****P < .0001.

Body composition was assessed by TD-NMR at 3 and 11 weeks of age (Fig. 3 and Table 2). At 3 weeks of age, BN and M520 males weighed the most and had the greatest fat-free mass compared with all other males (all comparisons with BN and M520 males *P* < .01, Fig. 3A and 3B). BN and M520 females weighed more than F344 females and ACI females (*P* < .01). ACI rats weighed the least and had the least fat-free mass compared with all other strains in both sexes (all comparisons with ACI *P*_Males_ < .001, *P*_Females_ < .01). Most strains had similar amounts of absolute fat mass at 3 weeks of age; however, ACI rats had the least amount of absolute fat mass compared with all other strains in both sexes (all comparisons with ACIs *P* < .0001, Fig. 3C). BN rats had lower relative fat mass than BUF, F344, M520, and WKY weanlings (BUF, F344, M520, and WKY comparisons with BN *P*_Males_ ≤ .09, *P*_Females_ < .01), but ACI rats had the lowest relative fat mass compared with all other strains in both sexes (all comparisons with ACI *P*_Males_ < .01, *P*_Females_ < .05, Fig. 3D).

**Table 2.** Body composition was assessed by time domain nuclear magnetic resonance at 3 and 11 weeks of age in male and female ACI, BN, BUF, F344, M520, and WKY rats.

	Males						Females					
	ACI	BN	BUF	F344	M520	WKY	ACI	BN*^a^*	BUF	F344	M520	WKY
**Week 3 body composition**												
Body weight (g)	32.8 ± 0.4 (25)	45.3 ± 0.9 (21)	40.0 ± 0.7 (21)	38.2 ± 0.8 (24)	46.0 ± 1.4 (24)	39.4 ± 1.0 (30)	31.1 ± 0.6 (26)	42.6 ± 1.1 (20)	40.2 ± 0.6 (20)	37.0 ± 0.7 (18)	43.2 ± 1.2 (20)	40.1 ± 0.6 (20)
Fat-free mass (g)	29.8 ± 0.3 (25)	40.3 ± 0.7 (21)	35.0 ± 0.6 (21)	33.2 ± 0.6 (24)	40.3 ± 1.1 (24)	34.5 ± 0.8 (30)	28.0 ± 0.5 (26)	37.8 ± 0.9 (20)	34.7 ± 0.5 (20)	31.8 ± 0.5 (18)	37.6 ± 0.9 (20)	34.4 ± 0.8 (20)
Fat mass (g)	3.1 ± 0.1 (25)	5.0 ± 0.2 (21)	5.1 ± 0.1 (21)	5.0 ± 0.2 (24)	5.7 ± 0.3 (24)	4.9 ± 0.3 (30)	3.0 ± 0.1 (26)	4.8 ± 0.3 (20)	5.5 ± 0.1 (20)	5.2 ± 0.2 (18)	5.6 ± 0.3 (20)	5.7 ± 0.3 (20)
Fat mass (%)	9.4 ± 0.3 (25)	11.0 ± 0.3 (21)	12.6 ± 0.2 (21)	12.8 ± 0.3 (24)	12.2 ± 0.4 (24)	12.1 ± 0.4 (30)	9.7 ± 0.2 (26)	11.1 ± 0.3 (20)	13.6 ± 0.1 (20)	14.0 ± 0.4 (18)	12.9 ± 0.4 (20)	14.0 ± 0.4 (20)
**Week 11 body composition**												
Body weight (g)	204.7 ± 3.0 (6)	225.9 ± 4.9 (7)	310.4 ± 3.3 (10)	267.0 ± 2.7 (12)	232.9 ± 5.6 (10)	261.5 ± 4.5 (11)	140.1 ± 1.3 (11)	144.8 ± 2.4 (10)	194.8 ± 3.1 (9)	156.0 ± 4.3 (7)	149.9 ± 2.5 (8)	181.1 ± 3.0 (9)
Fat-free mass (g)	187.4 ± 2.0 (6)	217.7 ± 4.3 (7)	255.8 ± 2.1 (10)	224.8 ± 2.0 (12)	215.0 ± 4.4 (10)	234.4 ± 3.7 (11)	130.4 ± 1.0 (11)	—	160.3 ± 3.0 (9)	135.8 ± 2.8 (7)	139.9 ± 1.8 (8)	162.5 ± 2.9 (9)
Fat mass (g)	17.7 ± 0.9 (6)	8.2 ± 1.1 (7)	54.6 ± 1.7 (10)	42.2 ± 2.1 (12)	17.9 ± 2.0 (10)	27.1 ± 1.5 (11)	9.7 ± 0.7 (11)	—	34.5 ± 1.1 (9)	20.2 ± 2.2 (7)	9.9 ± 1.1 (8)	18.7 ± 0.7 (9)
Fat mass (%)	8.6 ± 0.4 (6)	3.6 ± 0.4 (7)	17.6 ± 0.4 (10)	15.8 ± 0.7 (12)	7.6 ± 0.7 (10)	10.3 ± 0.4 (11)	6.9 ± 0.5 (11)	—	17.7 ± 0.5 (9)	12.8 ± 1.2 (7)	6.6 ± 0.6 (8)	10.3 ± 0.4 (9)

Mean ± SEM (n).

^*a*^Note that BN female body composition at 11 weeks of age is not shown because majority of BN females were not within the calibrated fat range of the TD-NMR instrument.

At 11 weeks of age, BUF males had the highest body weight compared with all other males (*P* < .0001), while BUF females were the highest compared with ACI, BN, F344, and M520 females (*P* < .001, Fig. 3E). ACI rats tended to be the lowest in body weight: ACI males weighed significantly less than all other males (*P* < .01), and ACI females weighed significantly less than BUF, F344, and WKY females (*P* < .05). Only body weight is shown for the BN females at 11 weeks of age because most BN females were below the TD-NMR instrument's calibrated range (<3.082 g total fat mass). The strain differences pattern in 11-week-old body weight was replicated in 11-week-old fat-free mass (Fig. 3F). ACI rats had significantly less fat-free mass than all other strains in both sexes (all comparisons with ACI *P*_Males_ < .01, *P*_Females_ < .01). BUF rats had the greatest absolute fat mass at 11 weeks of age compared with all other strains in both sexes (all comparisons with BUFs *P*_Males_ < .05, *P*_Females_ < .05), while BN males had the least of all males (*P* < .05, Fig. 3G). BUF and F344 males had significantly more percent fat mass compared with all other males at 11 weeks of age (*P* < .05, Fig. 3H). BUF females had the greatest relative fat mass compared with all other females (*P* < .05). BN males had the least fat mass as a relative of body weight compared with all other males (*P* < .05).

### Energy Homeostasis Includes Behavior

Metabolic cage data was analyzed from the 4- and 10-week time points to evaluate factors in energy homeostasis, like digestive efficiency (the proportion of calories absorbed from consumed food) and energy efficiency (body weight gained per calorie absorbed). The 24-hour fecal samples collected at both time points were analyzed by bomb calorimetry to determine fecal caloric density, and the results were used to calculate energy efficiency. There were no sex or strain differences in digestive efficiency at either time point (Fig. S3A and 3B (53, 59)). M520 females had the lowest energy efficiency compared with other females (*P* < .05) and M520 males had lower energy efficiency than F344 and WKY males (*P* < .001, Fig. S3C (53, 59)), suggesting that M520 rats expend more energy than other strains.

**Figure 4.**
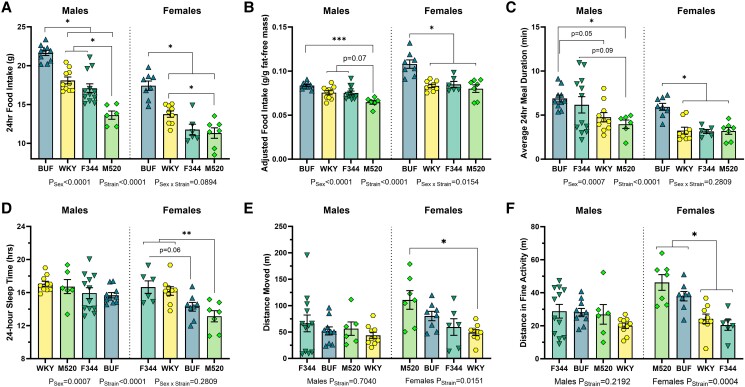
Eating behavior, sleep, and activity in male and female BUF, F344, M520, and WKY rats. At 11 weeks of age, animals were housed in the Promethion multiplexed metabolic phenotyping system. (A-C) Eating behavior was determined in 1 24-hour period including total food intake (A), food intake corrected for fat-free mass (B), and average meal duration (C). BUF rats consumed the most food compared with the other strains and had longer mealtimes. (D-F) Activity was determined in 1 24-hour period including total sleep time (D), distance travelled (E), and fine activity (F). M520 female rats slept less and had greater distance traveled and fine activity levels than WKY and F344 female rats. (A-D) Two-way ANOVA/Šídák's, *P < .05, **P < .01, ***P < .001. (E, F) Kruskal–Wallis/Dunn's, *P < .05. n = 6-12/sex/strain.

To determine whether other factors in energy homeostasis, like eating behavior, activity levels, and metabolic rate, vary in the strains, animals were housed in the Promethion multiplexed metabolic phenotyping system at 11 weeks of age. During the experiment, ACI and BN rats failed to acclimate to the Promethion housing system more often than the BUF, F344, M520, and WKY rats (<10 g fluid intake in 48 hours). Because of this high failure rate, ACI and BN data were excluded from this analysis. BUF rats consumed the most food compared with all other strains (all comparisons with BUF *P*_Males_ < .05, *P*_Females_ < .05, Fig. 4A, Table 3). M520 males consumed the lowest amount of food compared with the other males (*P* < .05), while M520 females consumed less food than BUF and F344 females (*P* < .05). Adjusting 24-hour food intake for fat-free body mass revealed that M520 males consume less food per gram of fat-free mass compared with BUF, F344, and WKY males (*P* ≤ .07) and that BUF females consume significantly more food per gram of fat-free mass compared with F344, M520, and WKY females (*P* < .05, Fig. 4B). The number of meals in 24 hours did not differ between males of different strains, but M520 females ate more meals in 24 hours than BUF females (*P* = .03, Table S3 (53)). The feeding rate (grams of food consumed per minute) was not different between the sexes or between the strains (Table S3 (53)). Meal duration was significantly different between strains (Fig. 4C). BUF females had a significantly longer meal duration than F344, WKY, and M520 females (*P* < .05). M520 males tended to have shorter meal durations than BUF and F344 males (*P* ≤ .09). These findings may indicate that the strains differ in satiety vs satiation mechanisms in controlling feeding behaviors.

**Table 3.** Energy homeostasis data collected in the Promethion in male and female BUF, F344, M520, and WKY rats

	Males				Females			
	BUF	F344	M520	WKY	BUF	F344	M520	WKY
24-hour food intake (g)	21.64 ± 0.34 (10)	17.09 ± 0.58 (12)	13.63 ± 0.53 (6)	18.10 ± 0.44 (10)	17.42 ± 0.60 (8)	11.76 ± 0.70 (6)	11.32 ± 0.68 (7)	13.76 ± 0.41 (9)
24-hour food per fat-free mass (g/g)	0.08 ± 0.00 (10)	0.08 ± 0.00 (12)	0.07 ± 0.00 (6)	0.08 ± 0.00 (10)	0.11 ± 0.01 (8)	0.09 ± 0.00 (6)	0.08 ± 0.00 (7)	0.08 ± 0.00 (9)
24-hour meal count	24 ± 1 (10)	23 ± 3 (12)	27 ± 1 (6)	24 ± 2 (10)	25 ± 3 (8)	31 ± 2 (6)	37 ± 5 (7)	28 ± 2 (9)
Food intake rate (g/min)	0.10 ± 0.00 (10)	0.13 ± 0.02 (12)	0.09 ± 0.02 (6)	0.12 ± 0.01 (10)	0.09 ± 0.01 (8)	0.10 ± 0.02 (6)	0.10 ± 0.02 (7)	0.11 ± 0.01 (9)
Meal duration (min)	6.89 ± 0.39 (10)	6.16 ± 0.93 (12)	3.97 ± 0.48 (6)	4.76 ± 0.52 (10)	5.94 ± 0.39 (8)	3.15 ± 0.22 (6)	3.20 ± 0.38 (7)	3.24 ± 0.39 (9)
24-hour sleep time (hr)	15.7 ± 0.3 (10)	15.9 ± 0.9 (12)	16.7 ± 0.9 (6)	17.1 ± 0.3 (10)	14.3 ± 0.5 (8)	16.7 ± 0.8 (6)	13.1 ± 0.7 (7)	16.2 ± 0.5 (9)
24-hour distance traveled (m)	52.1 ± 7.6 (10)	66.3 ± 15.9 (12)	56.4 ± 12.7 (6)	43.8 ± 5.7 (10)	80.3 ± 8.9 (8)	59.4 ± 15.7 (6)	110.7 ± 17.6 (7)	49.0 ± 5.5 (9)
24-hour fine activity (m)	28.2 ± 2.3 (10)	28.8 ± 4.1 (12)	26.9 ± 5.9 (6)	20.0 ± 1.6 (10)	37.9 ± 2.9 (8)	20.4 ± 3.2 (6)	46.2 ± 4.7 (7)	24.2 ± 2.9 (9)
Unaccounted calories (kcal/hr)	0.71 ± 0.06 (10)	0.32 ± 0.07 (12)	0.18 ± 0.06 (6)	0.23 ± 0.06 (10)	0.63 ± 0.04 (8)	0.20 ± 0.14 (6)	0.36 ± 0.06 (7)	0.19 ± 0.03 (9)
RMR (kcal/hr)	1.96 ± 0.04 (10)	1.61 ± 0.03 (12)	1.47 ± 0.05 (6)	1.69 ± 0.03 (10)	1.53 ± 0.04 (8)	1.45 ± 0.05 (6)	1.1 ± 0.03 (7)	1.37 ± 0.04 (9)
ANCOVA-Adj. RMR (kcal/hr)*^a^*	1.64 ± 0.04 (18)	1.45 ± 0.03 (18)	1.46 ± 0.04 (13)	1.53 ± 0.03 (19)	—	—	—	—

ACIs and BNs were excluded due to poor strain acclimation to the new cage environment. Data are mean ± SEM (n).

Abbreviations: ANCOVA, analysis of covariance; RMR, resting metabolic rate.

^*a*^Note that sex was not a significant factor in RMR adjustment. Sexes were pooled by strain, and strain ANCOVA-Adj. RMR values are placed in the male columns.

Activity measurements in the Promethion found that there were no significant strain differences in 24-hour sleep time in male rats (Fig. 4D and Table 3). In females, M520 rats slept less in 24 hours than F344 and WKY rats (*P* < .01). Consistent with the sleep time in males, there were no significant differences by strain in 24-hour distance traveled or in 24-hour fine activity (grooming, trembling, scratching, etc.) in male rats (Fig. 4E and 4F). M520 female rats showed greater 24-hour distance traveled than WKY females (*P* < .05), and M520 and BUF females had greater fine activity than F344 and WKY females (*P* < .05).

**Figure 5.**
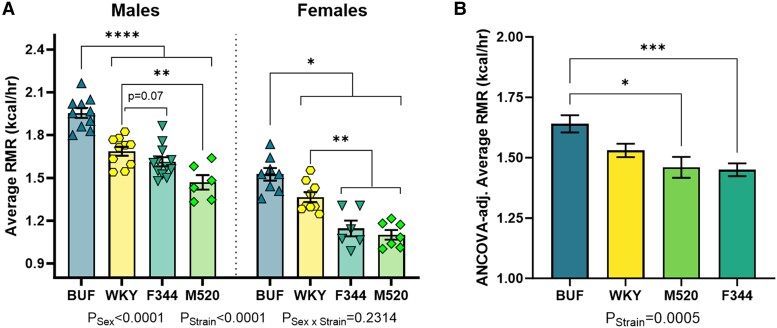
Resting metabolic rate in male and female BUF, F344, M520, and WKY rats. At 11 weeks of age, animals were housed in the Promethion multiplexed metabolic phenotyping system. Metabolic rate was determined over a 24-hour period. Average resting metabolic rate (A) was corrected for fat-free mass, fat mass, and 24-hour distance traveled (B). BUF rats had the highest resting metabolic rate compared with all other strains. (A) Two-way ANOVA/Šídák's, *P < .05, **P < .01, ****P < .0001. (B) Welch's 1-way ANOVA/Dunnett's by sex, *P < .05, ***P < .001. n = 6-12/sex/strain.

RMR during the lowest average 30-minute energy expenditure nadir in light hours of the 24-hour data collection period was measured. BUF rats showed the highest RMR compared with all other strains in both sexes (all comparisons with BUF *P*_Males_ < .0001, *P*_Females_ < .05, Fig. 5A and Table 3). After correction for influencing factors (fat-free mass, fat mass, and 24-hour distance traveled), BUF rats had higher RMR than F344 and M520 rats (*P* < .05, Fig. 5B and Table 3). Sex as a variable was tested in the RMR model, but sex was not included in the corrections for metabolic rate because the variable did not improve model fit. This result suggests that differences in fat-free mass, which includes muscle mass, are more determinant of RMR than sex. Because the Promethion estimates total energy expenditure via the measurement of aerobic metabolism, these results suggest that differences in protein and/or anaerobic metabolism may exist between the strains.

Unaccounted calories are the calories expended that are not attributable to heat production or growth but contribute to energy homeostasis (57). These calories were estimated based on the total calories consumed by food intake, the total energy expended during the Promethion week, body composition changes in the Promethion, and digestive efficiency in metabolic cages at 10 weeks of age. The BUF strain had higher unaccounted calories than F344, M520, and WKY rats in both sexes while there were no differences between the other strains (all comparisons with BUF *P*_Males_ < .001, *P*_Females_ ≤ .07, Fig. S4 (53), Table 3).

### Fat Distribution and Brown Adipose Modify BMI

BMI has for decades been the most accessible method for describing adiposity levels in humans. At 13 weeks of age, body length was measured and BMI was calculated (g body weight/m^2^ body length) (Fig. S5 (53, 59)). BUF males had the highest BMI; WKY males had the second highest BMI, followed by F344 males who had the third highest compared with all other males (all comparisons *P*_BUF_ < .0001, *P*_WKY_ < .0001, *P*_F344_ < .0001). BUF and WKY females had higher BMIs than all other females (all comparisons *P* < .0001).

**Figure 6.**
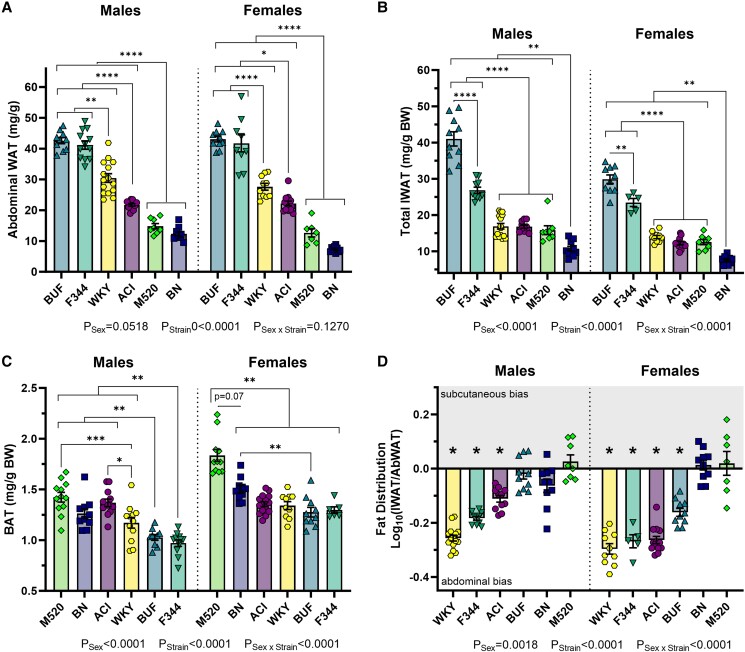
Fat distribution in male and female ACI, BN, BUF, F344, M520, and WKY rats. At 13 weeks of age, gonadal white adipose tissue and perirenal white adipose tissue (summed to calculate abdominal white adipose tissue [AbWAT]) (A), inguinal white adipose tissue (IWAT) (B), and brown adipose tissue (BAT) (D) were harvested, weighed, and corrected for body weight. BUF and F344 rats had the greatest amount of AbWAT compared with all other strains. BUF rats had the greatest amount of IWAT compared with all other strains. The log_10_ ratio of total IWAT to total AbWAT was calculated to estimate fat distribution. Negative values indicate a bias toward proportionally greater abdominal fat, while positive values indicate a bias toward proportionally greater subcutaneous fat (C). Male and female ACIs, F344s, and female BUF rats had proportionally greater AbWAT than IWAT. Male BUF, BN, and M520 rats had roughly equal proportion of AbWAT to IWAT. 2-way ANOVA/Šídák's, n = 9-13/sex/strain. *P < .05, **P < .01, ***P < .001, ****P < .0001.

To determine whether individual fat depots reflected the strain pattern in BMI data, GWAT, PWAT, IWAT, and BAT tissue samples were weighed and corrected for body weight. GWAT and PWAT were summed to estimate abdominal WAT (AbWAT). BUF and F344 rats had the greatest relative AbWAT compared with all other strains in both sexes (all comparisons *P*_BUF_ < .0001, *P*_F344_ < .0001, Fig. 6A). BN and M520 rats had the lowest AbWAT compared with all others strain in both sexes (all comparisons *P*_BN_ < .0001, *P*_M520_ < .0001). BUF rats had significantly more IWAT than all other strains in both sexes (all comparisons *P*_Males_ < .0001, *P*_Females_ < .01, Fig. 6B). F344 rats had more IWAT than ACI, BN, M520, and WKY rats in both sexes. Finally, BN rats had the least IWAT compared with all other strains in both sexes (all comparisons *P*_Males_ < .01, *P*_Females_ < .01). Interestingly, M520 rats usually had the most BAT compared with other strains: M520 males had more BAT than WKY, BUF, and F344 males (*P* < .001), while M520 females had the most BAT for all females (*P* ≤ .07, Fig. 6C). BUF and F344 rats tended to have the least BAT compared with most strains.

Considerable evidence now suggests that BMI poorly indicates obesity status since it does not capture fat storage in individual depots (60). To describe whether the animals had proportionally more abdominal or subcutaneous fat, the ratio of AbWAT to IWAT was calculated, tested to see whether the distribution was different from a theoretical ratio of 1, and plotted as a Log_10_ transformation (Fig. 6D). WKY rats, F344 rats, ACI rats, and BUF females had fat distribution values less than 1 (*P* < .001), indicating proportionally more AbWAT than IWAT.

### Endocrine Tissues and Circulating Hormones Reveal Thyroid Disease Potential

**Figure 7.**
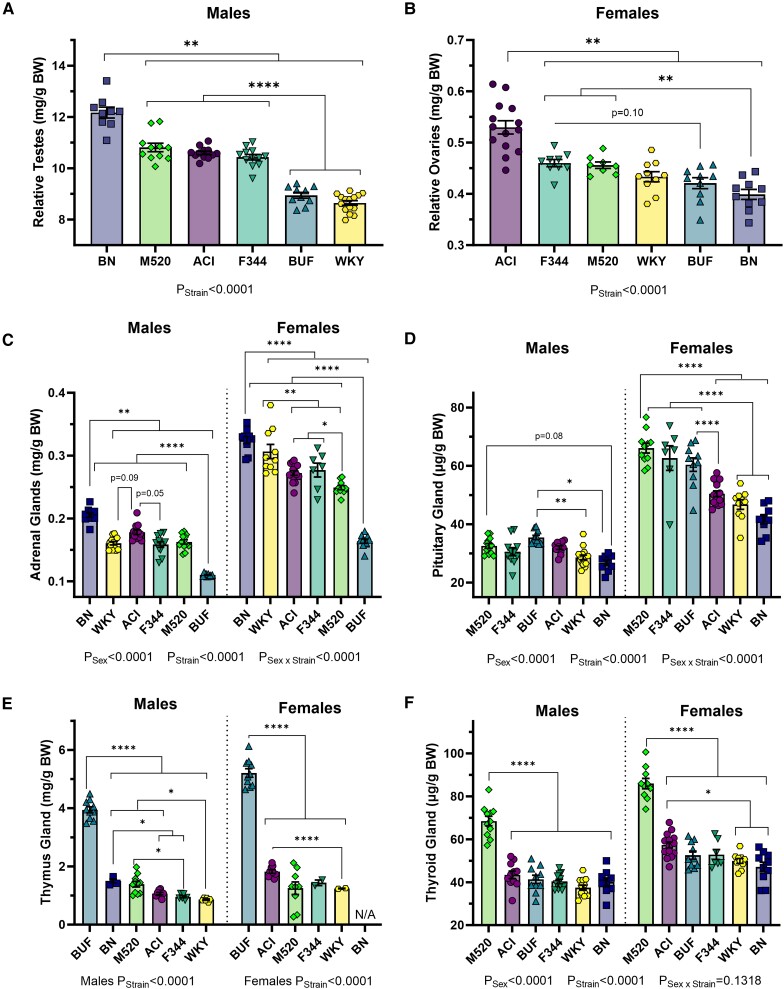
Endocrine tissue weights in male and female ACI, BN, BUF, F344, M520, and WKY rats. At 13 weeks of age, gonads (testes (A), ovaries (B)), adrenal (C), pituitary (D), thymus (E), and thyroid (F) were harvested, weighed, and corrected for body weight. BN males have significantly greater relative testes weight than all other males. ACI females have significantly higher relative ovaries weight than all other females. BN rats tended to have the greatest adrenal weight and least pituitary weight per gram body weight. BUF rats had the greatest thymus weight compared with all other strains. M520 rats had the greatest thyroid weight compared with all other strains. (C, D, F) Two-way ANOVA/Šídák's. (A, B, E) Welch's 1-way ANOVA/Dunnett's by sex, n = 2-15/sex/strain. *P < .05, **P < .01, ****P < .0001.

Metabolic disorders are significantly influenced by the endocrine system (61), so we surveyed endocrine gland weights (gonads, adrenal glands, pituitary gland, thymus gland, thyroid gland) across rat strains at 13 weeks of age and corrected for body weight. BN males have significantly greater relative testes weight compared with all other males (*P* < .01, Fig. 7A). BUF and WKY males had significantly lower relative testes weight than ACI, BN, F344, and M520 males (*P* < .0001). ACI females had significantly higher relative ovaries weight than all other females (*P* < .01, Fig. 7B), while BN females had significantly lower relative ovary weight than ACI, F344, and M520 females (*P* < .01). In both sexes, BN rats had the highest relative adrenal weight compared with all other strains (all comparisons *P*_Males_ < .01, *P*_Females_ < .0001, Fig. 7C). BUF rats had the lowest relative adrenal weight compared with all other strains in both sexes (all comparisons *P*_Males_ < .0001, *P*_Females_ < .0001). Relative pituitary weight did not vary appreciably in male rats (Fig. 7D). In females, BN and WKY rats had significantly lower relative pituitary weight than BUF, F344, and M520 females (*P* < .0001). F344 females had greater relative pituitary weight than ACI, BN, and WKY females (*P* < .0001). BUF rats had considerably higher relative thymus weight than all other strains (all comparisons *P*_Males_ < .0001, *P*_Females_ < .0001, Fig. 7E). BN males had significantly more relative thymus mass than ACI, F344, and WKY males (*P* < .05). M520 rats showed high relative thyroid weight compared with all other strains in both sexes (all comparisons *P*_Males_ < .0001, *P*_Females_ < .0001, Fig. 7F).

**Figure 8.**
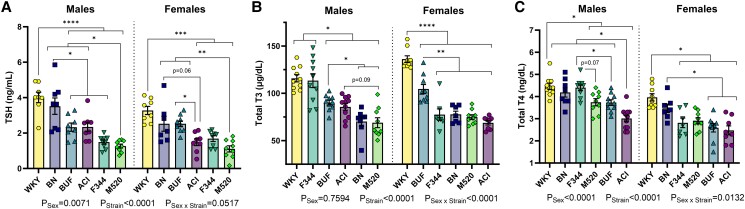
Levels of hypothalamic–pituitary–thyroid axis hormones in male and female ACI, BN, BUF, F344, M520, and WKY rats. TSH (A), total T3 (B), and total T4 (C) levels were measured in serum collected at 13 weeks of age. WKY rats tended to have the greatest levels of TSH, total T3, and total T4 compared with most other strains. M520 rats tended to have the lowest TSH levels. Two-way ANOVA/Šídák's, n = 7-11/sex/strain. *P < .05, **P < .01, ***P < .001, ****P < .0001.

To determine whether thyroid weight differences associated with circulating hypothalamic–thyroid–pituitary axis hormone levels, serum rat TSH, total T3, and total T4 were assessed at 13 weeks of age (Fig. 8 and Table 4). WKY males had higher TSH levels than ACI, BUF, F344, and M520 males (*P* < .0001), and WKY females had higher TSH levels than ACI, F344, and M520 females (*P* < .001, Fig. 8A). BNs had comparable levels of TSH to WKYs in both sexes. M520 males had the lowest TSH levels compared with ACI, BN, BUF, and WKY males (*P* < .05), and M520 females had lower TSH levels than BN, BUF, and WKY females (*P* < .01). WKY and F344 males had significantly higher total T3 than all other males (all comparisons *P* < .05); however, WKY females had the highest total T3 compared with females of all other strains (*P* < .0001, Fig. 8B). BUF females had higher total T3 than ACI, BN, F344, and M520 females (*P* < .01). Total T4 in WKY males was higher than ACI, BUF, and M520 males (*P* < .05) and in WKY females were higher than ACI, BUF, F344, and M520 females (*P* < .05, Fig. 8C). ACI rats had lower total T4 than most strains in both sexes: ACI had the lowest total T4 of all males (*P* < .05), and ACI females had lower total T4 than BN and WKY females (*P* < .05).

**Table 4.** Circulating thyroid stimulating hormone (TSH), total triiodothyronine (tT3), and total thyroxine (tT4) were measured in serum at 13 weeks of age in male and female ACI, BN, BUF, F344, M520, and WKY rats.

		Serum TSH (ng/dL)	Serum tT3 (µg/dL)	Serum tT4 (ng/dL)
Males	ACI	2.3 ± 0.3 (8)	85.6 ± 3.5 (11)	3.0 ± 0.1 (11)
	BN	3.5 ± 0.5 (8)	70.2 ± 4.8 (8)	4.2 ± 0.2 (8)
	BUF	2.3 ± 0.2 (8)	90.3 ± 2.6 (10)	3.7 ± 0.1 (10)
	F344	1.5 ± 0.1 (9)	113.4 ± 7.4 (10)	4.4 ± 0.1 (10)
	M520	1.3 ± 0.1 (9)	68.9 ± 5.3 (9)	3.7 ± 0.2 (9)
	WKY	4.0 ± 0.3 (8)	115.9 ± 3.9 (10)	4.5 ± 0.1 (10)
Females	ACI	1.5 ± 0.2 (8)	68.8 ± 2.4 (8)	2.5 ± 0.2 (8)
	BN	2.5 ± 0.4 (7)	77.7 ± 3.4 (7)	3.5 ± 0.2 (7)
	BUF	2.5 ± 0.2 (9)	104.5 ± 4.8 (9)	2.6 ± 0.2 (9)
	F344	1.7 ± 0.2 (8)	77.4 ± 6.1 (7)	2.8 ± 0.2 (7)
	M520	1.1 ± 0.2 (9)	74.8 ± 2.5 (9)	2.9 ± 0.1 (9)
	WKY	3.3 ± 0.2 (8)	136.1 ± 3.5 (8)	4.0 ± 0.2 (9)

Data are mean ± SEM (n).

### Expression of Adaptive Thermogenesis and IWAT Beiging Genes Vary Across Strains

**Figure 9.**
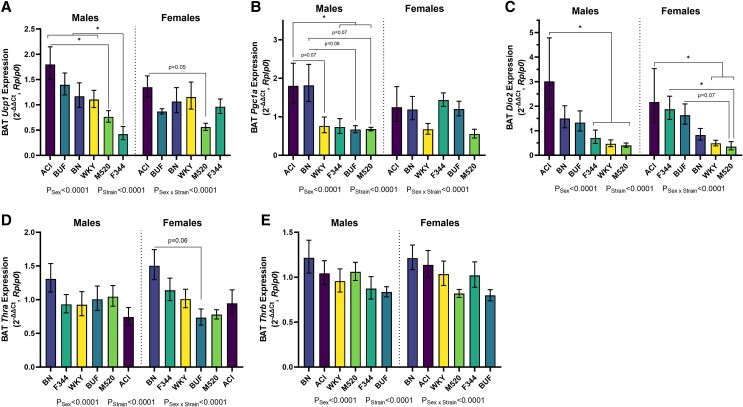
Gene expression of adaptative thermogenesis markers and thyroid hormone receptors in BAT in male and female ACI, BN, BUF, F344, M520, and WKY rats. Expression of *Ucp1* (A), *Ppargc1a* (B), *Dio2* (C), *Thra* (D), and *Thrb* (E) was determined by RT-qPCR in BAT samples. F344 males had the lowest expression of *Ucp1* compared with most other males. ACI males tended to have the highest expression of *Ppargc1a* and *Dio2* compared with other males. M520 rats tended to have the lowest expression of *Ucp1*, *Ppargc1a*, and *Dio2*. Note that 2^−ΔΔCt^ values are calculated against the average of all strains within each sex. Two-way ANOVA/Šídák's; n = 7-8/sex/strain. *P < .05.

Thyroid hormones and their receptors help control adaptive thermogenesis gene expression in BAT as well as beiging in subcutaneous WAT (62, 63). To determine strain differences in adaptive thermogenesis gene expression, thyroid hormone receptors (*Thra*, *Thrb*), an enzyme that converts thyroid hormone to its active state (*Dio2*), the transcription factor Pgc1α that directly regulates *Ucp1* (*Ppargc1a*), and *Ucp1* were measured by RT-qPCR in BAT samples (Fig. 9). F344 males had significantly lower expression of *Ucp1* than ACI, BN, BUF, and WKY males (*P* < .05, Fig. 9A). *Ppargc1a* was significantly upregulated in ACI males compared with BUF, F344, M520, and WKY males (*P* ≤ .07, Fig. 9B). ACI males had upregulated *Dio2* compared with F344, M520, and WKY males (*P* < .05, Fig. 9C). Females showed few differences in *Ucp1* and *Pgc1a* expression, but M520 females showed downregulated *Dio2* compared with ACI, BUF, and F344 females (*P* ≤ .07). There were few differences in *Thra* and *Thrb* expression across the strains in both sexes (Fig. 9D and 9E).

To determine whether strains had differences in subcutaneous WAT beiging potential, *Ucp1* was measured by RT-qPCR in IWAT samples (Fig. S6 (53)). ACI males had dramatically higher expression of *Ucp1* than all other males (average ΔCt of BN, BUF, F344, M520, and WKY = 21.37, average ΔCt of ACI = 6.96, all comparisons with ACI *P* < .0001). In females, the ACI and M520 strains had higher *Ucp1* expression than the BN and WKY strains (BN and WKY comparisons to ACI *P* ≤ .08, BN and WKY comparisons with M520 *P* < .05).

## Discussion

**Figure 10.**
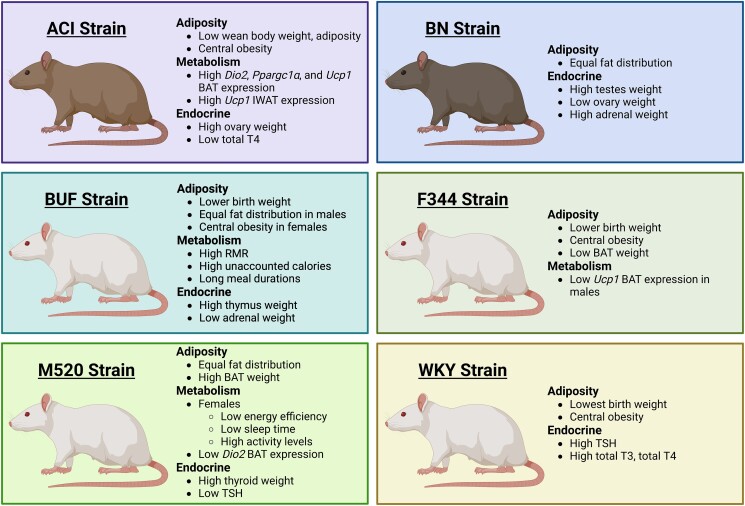
Summary list of new adiposity, metabolism, and endocrine health traits in ACI, BN, BUF, F344, M520, and WKY rat strains. Created using BioRender.com.

The use of animal models that demonstrate genetic diversity is an important approach to improve the translatability of laboratory animal studies to the human population. We studied the growth, energy homeostasis, and endocrine glands of 6 substrains similar to the HS rat founder strains to broaden the health profiles of the HS rat founders. This report combined novel observations made in breeding colonies with new analyses of previously published data of contemporaneous controls from Wagner et al (48) and the results of new experiments in banked tissues. Our results highlight that the reanalysis of available data can provide valuable scientific insight without the use of additional animals as justified by the Replacement, Reduction, and Refinement IACUC guidelines. Our data show highly variable metabolic and endocrine health traits in ACI, BN, BUF, F344, M520, and WKY rat strains (Fig. 10). This is the first report of numerous strain- and sex-specific traits and comparisons of birth weight, physical activity, endocrine gland weights, and *Ucp1* gene expression in BAT and IWAT in animals similar to the HS rat founders. Our study also identified the HS rat population as a novel model to investigate genetic contributions to early life adversity and adrenal and thyroid pathophysiology.

### ACI Strain

The ACI strain had the lowest body weight gain from 1 to 13 weeks of age compared with the other strains. There was a noticeable inflection point early in the ACI growth curve, and ACI weanlings at 3 weeks of age had the lowest body weight, the lowest absolute total body fat mass, and the lowest relative total body fat mass compared with the other strains. ACI weanlings may have lower body weight and fat mass as a result of maternal behavior, abnormal changes in milk quality late in lactation, or premature weaning from milk. Lactation is a thermoregulatory challenge for rat dams (64), so it is possible that lactating ACI dams move away from the litter to improve heat dissipation. A recent study reported that much like human milk, rat milk has important immunoglobulin, adipokine, and growth factor content that changes over the lactation period (65). Premature weaning may negatively affect postnatal development as the pups would lose access to these factors in milk (66). Lactation is recognized as a critical programming window for metabolic syndrome (67), and malnourishment in childhood can negatively affect metabolic health in adulthood (68). In young adulthood, ACI rats had a low BMI, but the strain had proportionally more AbWAT than IWAT in their fat distribution, indicating a central obesity phenotype. Other publications have noted that adult ACI males have high fasted glucose, a low insulin response to glucose challenge, and decreased pancreatic beta cell function (19). These data demonstrate that the ACI strain exhibits multiple poor metabolic health traits in adulthood, further supporting the possibility that ACI rats experience malnourishment before weaning.

ACIs also tended to have lower total T4 than other strains. Previous studies show that ACI rats have lower T3 sulfonation than F344 and SD rats (69), suggesting a strain difference in thyroid hormone metabolism. In BAT, ACI males tended to have the highest gene expression of *Dio2*, encoding the enzyme deiodinase II that converts the less active T4 to the more active T3 in target tissues. This may indicate that the ACI strain has a greater capacity for thyroid hormone signaling to regulate the BAT transcriptome. ACI males had significantly higher expression of *Ucp1* in IWAT than all other males, a sign that ACI males have a greater capacity for subcutaneous WAT beiging (62, 63). Metabolic rate in the ACI strain may show effects from an increase in thyroid hormone sensitivity and in high *Ucp1* expression; however, these questions must be addressed in a subsequent study because we were unable to assess ACI metabolic rate using the Promethion. Future studies will determine whether the ACI strain displays increased tissue responses to thyroid hormone.

### BN Strain

The BN rat was a strain that had low body weight during adulthood despite having a higher weaning weight. Male and female BNs had lower percent fat mass than other strains (except ACIs) at weaning and had the lowest percent fat mass and individual fat depot weight in adulthood compared with the other rat strains. The BN fat distribution was similar between AbWAT and IWAT and their BMI was low. Low fat mass as well as extensive reproductive dysfunction are known in the BN strain (17, 27, 28). In this study, we found that BN males had the greatest relative testes weight, and the BN females had the lowest relative ovaries weight. It is probable that these abnormal gonad weights are part of the BN strain reproductive dysfunction: testes hypertrophy is likely occurring to maximize steroidogenesis, and decreased ovarian weight may contribute to hormone imbalance or low litter size.

Interestingly, the BN strain had a ∼33% higher relative adrenal weight than all other strains. This is the first report of increased adrenal weight in the BN strain. A previous study investigating adrenal function in chronic stress did not find a difference in male BN, Fischer, and WKY adrenal weights (70), which is inconsistent with our study. BN males have shown resistance to the suppressive effects of dexamethasone treatment on basal corticosterone levels (71), suggesting that BNs may have some resistance to adrenocorticotropin signaling and adrenal dysfunction. Glucocorticoids secreted from the adrenal glands help control reproductive functions by affecting central and peripheral tissues in the hypothalamic–pituitary–gonadal axis (72). In underweight women, congenital adrenal hyperplasia and ovarian dysfunction are common causes of reproductive disorders (73). High adrenal weight in the BN strain may reflect high circulating glucocorticoids that could contribute to poor reproduction in the BN. It is possible that the adrenal glands are an unrecognized component of BN reproductive dysfunction. Future investigations will define the significance of glucocorticoids on BN reproductive health.

### BUF Strain

Our data are consistent with reports of extremely high thymus weight in BUF rats (over triple the average weight in the other strains) (33). The BUF strain had the lowest relative adrenal weight compared with all other strains, the first report of low adrenal weight in this strain. Given that thymus weight is an index of circulating glucocorticoids (74), it is probable that the BUF strain has low circulating glucocorticoids reflecting hypotrophy of the adrenal glands. The BUF strain was unique in the birth weight analysis because it was the only strain lacking correlations between average birth weight and litter size at birth in both sexes, meaning that pups born in smaller litters do not have higher birth weights than pups born in large litters. The absence of the common relationship between birth weight and litter size may reflect some form of restricted in utero growth. Autoimmune disorders are commonly observed in BUF rats with age, including hyperplasia of the peripheral T cell system (32, 34, 75, 76). Glucocorticoids control immune functions in part by influencing T cell homeostasis (77). T cell functions are critical for healthy pregnancies, and decreased CD8(+) T cell infiltration into the placenta can cause fetal growth restriction (78, 79). Additional observations in BUF colonies are needed to determine the factors influencing BUF birth weight and how the BUF maternal immune system changes in pregnancy.

Despite being slightly smaller at birth, BUF males and females had the highest body weight gained throughout the study, had high adiposity levels by both body composition measurements and individual fat depot weight, and had high BMI. These data are all consistent with previous reports in the BUF strain (17, 19). Considering relative IWAT weight in fat distribution revealed that while the BUF strain has high adiposity, the distribution of adipose between abdominal and subcutaneous depots in males is equivalent and in females it is skewed toward greater abdominal fat. This suggests that BUF males have a general obesity phenotype while females have a central obesity phenotype. Male and female BUF rats consumed the most calories that were unaccounted for by aerobic heat production, suggesting that the BUF rats have greater energy needs to support nonaerobic metabolism in the gut microbiome or in other locations (57). BUF rats had greater average meal durations than F344, M520, and WKY rats but similar food intake rates, which may explain the overall high food intake and the high adiposity levels observed in this strain. Previous studies show that male BUF rats have high fasting insulin and a high insulin response to glucose challenge (19), but no studies have investigated ghrelin and leptin, hormones that are critical regulators of hunger and satiety (80, 81). Future studies will determine whether dysregulated ghrelin and leptin differentially contribute to the fat distributions in male and female BUF rats.

### F344 Strain

Many observations made in this study on the F344 strain are consistent with previous reports of increased adiposity (17, 19, 82). In adulthood, male and female F344 rats had high adiposity levels as measured by body composition and individual fat depot weights as well as a modest to high BMI. Fat distribution in male and female F344 rats showed proportionally greater AbWAT than IWAT, indicating a central obesity phenotype. Here we identified novel adiposity phenotypes in F344 rats. F344 rats tended to have little relative BAT mass in both sexes, which is in line with their larger white adipose depots. Male F344 rats also had lower expression of *Ucp1* in BAT than other males, a novel finding that is consistent with high adiposity and central obesity. F344 pups are smaller at birth than ACI, BN, and M520 pups and have similar birth weights as BUF rats, another strain used as an obesity model. This is the first report of lower birth weight in F344 pups than other lean rat strains. Intrauterine life is a critical programming period for adult metabolic health (83). Future studies will investigate if early life programming contributes to lower birth weight in F344 pups and to their high adiposity gain in adulthood.

### M520 Strain

M520 pups were comparatively heavier at birth and were an average body weight at weaning, yet in adulthood they had low body weight gain in both sexes. M520 rats also had lower adiposity at 11 weeks of age by body composition, at 13 weeks of age by weight of individual fat depots, and in BMI, which are consistent with observations made in previous studies (17, 19). M520s also have equal proportions of AbWAT and IWAT, indicating a relatively healthy fat distribution. Female M520s, in fact, had more relative BAT mass than all other females, which is the first report of high BAT weight in the M520 strain. M520 females but not males slept less and tended to have higher activity levels than other strains. These observations are consistent with the M520 females having the lowest energy efficiency of all females: calories consumed by M520 females support higher activity rather than body growth.

Relative thyroid weight was ∼67% greater in M520s than all other strains in both sexes. This is the first report of high thyroid weight in the M520 strains. The M520 strains appear to be euthyroid at 13 weeks of age based on their total T3 and T4 levels; however, M520 TSH tended to be the lowest in both sexes. These data suggest that the M520 strain, or at least the M520 females, may be on the edge of subclinical hyperthyroidism. In humans, subclinical hyperthyroidism may be caused by Graves disease or nodular goiter (84). Also, hyperthyroidism is a common cause of sleep dysfunction in humans, including prolonged sleep latency, trouble maintaining sleep, daytime sleepiness, and even restless leg syndrome (85). Using the Promethion, we found evidence of decreased 24-hour sleep time in the M520 females as well as both forms of activity, ambulation and fine activity, all of which appear consistent with sleep disturbance noted in hyperthyroidism. Untreated hyperthyroidism can also lead to tremors and weight loss (86), which is again consistent with increased fine activity, low body weight, and low adiposity in M520 females. Increased food intake is seen in rats with hyperthyroidism (87); however, there was no evidence of increased food intake by M520 rats compared with other strains. Current models for Graves disease rely on induction methods using repetitive vaccinations using adenovirus, in vivo electroporation of a thyrotropin receptor–containing plasmid, or thyroid hormone supplementation (88, 89). Therefore, the M520s may be a novel model of mild hyperthyroidism. Future studies in the M520 strain will investigate thyroid function and energy homeostasis in young and aged rats.

### WKY Strain

Birth weights of WKY pups were the lowest of all strains, with both male and female WKYs being ∼15% lower than ACI, BN, and M520 pups and ∼7% lower than BUF and F344 pups. This is the first study comparing WKY birth weights to leaner rat strains not derived from the Wistar rat. Adiposity levels in the WKYs are consistent with previous reports (17, 19). Male and female WKY rats have a central obesity fat distribution with proportionally greater AbWAT than IWAT. Previous studies reported that the WKY males have elevated TSH levels and normal thyroid hormone levels (90), which is consistent with our data. No report on female WKY TSH or thyroid hormone levels exist. Our study is the first to report high TSH level in WKY females. The WKY strain is a well-studied rat model for hypothalamic–pituitary–adrenal axis dysfunction and subclinical hypothyroidism, and the WKY strain has a genetic risk for diabetes susceptibility (40‐42, 90, 91). Hypothalamic–pituitary–adrenal axis dysfunction is a common occurrence in humans with low birth weight (92). Low birth weight individuals have an increased risk for type II diabetes development (93), and extremely low birth weight individuals can show lasting DNA methylation changes in genes controlling hypothalamic–pituitary–adrenal axis function, adrenal hormone regulation, and cardiometabolic disease risk (94). Interestingly, a meta-analysis found that mothers with subclinical hypothyroidism during pregnancy had a higher risk of small for gestational age and low birth weight babies (95). Therefore, the lower birth weights observed in the WKY strain likely reflect their genetic risks for hypothalamic–pituitary–adrenal axis dysfunction and cardiometabolic disease. Future investigations will study the complex physiological contributions to low birth weight in WKY pregnancies.

### Limitations

One major limitation of this comprehensive study was that the ACI and BN strains failed to acclimate to the Promethion system by drinking insufficient fluid, making reporting on energy expenditure and activity impossible in these strains. It is odd that rats would not drink freely available fluid resulting in self-inflicted acute dehydration. The Promethion system uses an infrared photobeam grid interruption approach to assess activity, so the paper enrichment the rats were accustomed to having in their home cages was not allowed in the Promethion. It is possible that the lack of environmental enrichment presented a thermoregulatory challenge to the ACI and BN strains and, subsequently, the rats altered their drinking behavior. Our labs have observed in unpublished data that ambient temperature modifies fluid and food intake behaviors in rodents. Additional studies are required to determine whether cage architecture or ambient temperature and humidity changes improve fluid intake by ACI and BN strains in the Promethion system. Another limitation of this study was that blood was collected at only 1 time point. Circulating thyroid related hormones have circadian fluctuations, such as the nocturnal surge between 02:00 and 04:00 in humans and a nadir around 12:00 (96). There may be differences in TSH, total T3, and total T4 levels between ACI, BN, BUF, F344, M520, and WKY rats that were not captured due to the selected time point. Future studies assessing thyroid related hormone levels will include a comprehensive panel of time points to capture circadian variation.

As mentioned in the introduction, we were able to study 6 substrains that are genetically similar to the HS founder N substrains. The MR and WN strains were not studied since they are not yet cryo-resuscitated or are extinct, respectively. High genetic identity is shared between the ACI/EurMcwi, BN/NHsdMcwi, M520/NRrrcMcwi, and WKY/NCrl substrains used here and the HS rat founder N substrains (97.1-99.9%), except for the BUF substrain (BUF/MnaMcwi shares 73.6% genetic identity with BUF/N) (47). There are F344 substrains with high genetic identity to F344/N, but the F344/StmMcwi has not been assessed. Even though we were unable to study the exact HS rat founder substrains, we submit that the high genetic similarities between the studied substrains and the HS rat founders increase the likelihood that strain-specific energy homeostasis and endocrine traits identified here were present in the N substrains and inherited by the HS rat population.

In summary and conclusion, we found that 6 genetically diverse substrains similar to the HS rat founders exhibit a wide range of endocrine and metabolic health states. We report many new traits that contribute to commonly reported adiposity and metabolic phenotypes and inform adult disease risks. Our birth weight comparisons provide additional context for early life programming that influences metabolic health in adulthood. Our fat distribution data showed that adiposity location rather than amount better mirrors metabolic health status. Our adrenal weight data postulates circulating glucocorticoid variation across strains that may affect multiple body systems and influence overall health. Our compelling thyroid weight data and HPT axis hormone levels suggest that the HS rat population may have unrealized potential for genetic mapping of both hypothyroid and hyperthyroid diseases. The HS rat population likely harbors risk alleles for the strain-specific traits identified in this study, making the HS rat a powerful tool to investigate interventions on endocrine and metabolic health.

## Data Availability

Metabolic phenotyping data from body weights, body composition, metabolic cages, and blood analytes as well as tissue weights are publicly available through the Rat Genome Database (https://rgd.mcw.edu/, with unique accession RGD:401827281).

## Abbreviations

AbWAT: abdominal white adipose tissue
ACI: August Copenhagen Irish
AGD: anogenital distance
ANOVA: analysis of variance
BAT: brown adipose tissue
BMI: body mass index
BN: Brown Norway
BUF: Buffalo
CV: coefficient of variation
F344: Fisher 344
GWAT: gonadal white adipose tissue
HS: Heterogeneous Stock
IWAT: inguinal white adipose tissue
M520: Marshall 520
MR: Maudsley Reactive
PWAT: perirenal white adipose tissue
RIA: radioimmunoassay
RMR: resting metabolic rate
RT-qPCR: reverse transcription quantitative polymerase chain reaction
T3: triiodothyronine
T4: thyroxine
TD-NMR: time domain nuclear magnetic resonance
TSH: thyroid-stimulating hormone
WKY: Wistar/Kyoto
WN: Inbred Wistar

## References

[1] Adult Obesity Prevalence Maps . National Center for Chronic Disease Prevention and Health Promotion, Division of Nutrition, Physical Activity, and Obesity. Updated 27 September 2022. Accessed 4/25/2023.

[2] National Health and Nutrition Examination Survey 2017–March 2020 Prepandemic Data Files Development of Files and Prevalence Estimates for Selected Health Outcomes, 10.15620/cdc:106273 (2021). https://stacks.cdc.gov/view/cdc/106273

[3] Dai H, Alsalhe TA, Chalghaf N, Riccò M, Bragazzi NL, Wu J. The global burden of disease attributable to high body mass index in 195 countries and territories, 1990-2017: an analysis of the global burden of disease study. PLoS Med. 2020;17(7):e1003198. 32722671 10.1371/journal.pmed.1003198 PMC7386577

[4] World Obesity Atlas 2023. World Obesity Federation; https://data.worldobesity.org/publications/?cat=19

[5] Maes HH, Neale MC, Eaves LJ. Genetic and environmental factors in relative body weight and human adiposity. Behav Genet. 1997;27(4):325‐351. 9519560 10.1023/a:1025635913927

[6] Stunkard AJ, Foch TT, Hrubec Z. A twin study of human obesity. JAMA. 1986;256(1):51‐54. 3712713

[7] Heindel JJ, Howard S, Agay-Shay K, et al Obesity II: establishing causal links between chemical exposures and obesity. Biochem Pharmacol. 2022;199:115015. 35395240 10.1016/j.bcp.2022.115015 PMC9124454

[8] Coelho M, Oliveira T, Fernandes R. Biochemistry of adipose tissue: an endocrine organ. Arch Med Sci. 2013;9(2):191‐200. 23671428 10.5114/aoms.2013.33181 PMC3648822

[9] Tchen R, Tan Y, Boyd Barr D, et al Use of high-resolution metabolomics to assess the biological perturbations associated with maternal exposure to bisphenol A and bisphenol F among pregnant African American women. Environ Int. 2022;169:107530. 36148711 10.1016/j.envint.2022.107530 PMC9664380

[10] Ylli D, Sidhu S, Parikh T, Feingold KR, Anawalt B, Blackman MR et al Endocrine Changes in Obesity. In: Feingold KR Anawalt B Blackman MR, et al (ed.), Endotext [Internet]. South Dartmouth (MA): MDText.com; 2022. Updated September 6, 2022. PMID: 25905281.

[11] Gordon-Larsen P, French JE, Moustaid-Moussa N, et al Synergizing mouse and human studies to understand the heterogeneity of obesity. Adv Nutr. 2021;12(5):2023‐2034. 33885739 10.1093/advances/nmab040 PMC8483969

[12] Hansen C, Spuhler K. Development of the national institutes of health genetically heterogeneous rat stock. Alcohol Clin Exp Res. 1984;8(5):477‐479. 6391259 10.1111/j.1530-0277.1984.tb05706.x

[13] Saar K, Beck A, Bihoreau MT, et al SNP And haplotype mapping for genetic analysis in the rat. Nat Genet. 2008;40(5):560‐566. 18443594 10.1038/ng.124 PMC5915293

[14] Woods LC, Mott R. Heterogeneous stock populations for analysis of complex traits. Methods Mol Biol. 2017;1488:31‐44. 27933519 10.1007/978-1-4939-6427-7_2 PMC5869698

[15] Chitre AS, Polesskaya O, Holl K, et al Genome-wide association study in 3,173 outbred rats identifies multiple loci for body weight, adiposity, and fasting glucose. Obesity (Silver Spring). 2020;28(10):1964‐1973. 32860487 10.1002/oby.22927 PMC7511439

[16] Hong-Le T, Crouse WL, Keele GR, et al Genetic mapping of multiple traits identifies novel genes for adiposity, lipids, and insulin secretory capacity in outbred rats. Diabetes. 2023;72(1):135‐148. 36219827 10.2337/db22-0252 PMC9797320

[17] Keele GR, Prokop JW, He H, et al Genetic fine-mapping and identification of candidate genes and variants for adiposity traits in outbred rats. Obesity (Silver Spring). 2018;26(1):213‐222. 29193816 10.1002/oby.22075 PMC5740008

[18] Tsaih SW, Holl K, Jia S, et al Identification of a novel gene for diabetic traits in rats, mice, and humans. Genetics. 2014;198(1):17‐29. 25236446 10.1534/genetics.114.162982 PMC4174929

[19] Solberg Woods LC, Holl KL, Oreper D, Xie Y, Tsaih SW, Valdar W. Fine-mapping diabetes-related traits, including insulin resistance, in heterogeneous stock rats. Physiol Genomics. 2012;44(21):1013‐1026. 22947656 10.1152/physiolgenomics.00040.2012 PMC3524769

[20] Deal AW, Seshie O, Lenzo A, Cooper N, Ozimek N, Solberg Woods LC. High-fat diet negatively impacts both metabolic and behavioral health in outbred heterogeneous stock rats. Physiol Genomics. 2020;52(9):379‐390. 32687430 10.1152/physiolgenomics.00018.2020 PMC7509248

[21] Crouse WL, Das SK, Le T, et al Transcriptome-wide analyses of adipose tissue in outbred rats reveal genetic regulatory mechanisms relevant for human obesity. Physiol Genomics. 2022;54(6):206‐219. 35467982 10.1152/physiolgenomics.00172.2021 PMC9142160

[22] Samanas NB, Commers TW, Dennison KL, et al Genetic etiology of renal agenesis: fine mapping of renag1 and identification of kit as the candidate functional gene. PLoS One. 2015;10(2):e0118147. 25693193 10.1371/journal.pone.0118147 PMC4333340

[23] Solleveld HA, Boorman GA. Spontaneous renal lesions in five rat strains. Toxicol Pathol. 1986;14(2):168‐174. 3764314 10.1177/019262338601400204

[24] Ohshima M, Yamahara K, Ishikane S, et al Systemic transplantation of allogenic fetal membrane-derived mesenchymal stem cells suppresses Th1 and Th17 T cell responses in experimental autoimmune myocarditis. J Mol Cell Cardiol. 2012;53(3):420‐428. 22796574 10.1016/j.yjmcc.2012.06.020

[25] Taguchi O, Kontani K, Ikeda H, Matsuyama M. An intrinsic thymic epithelial abnormality is responsible for the spontaneous development of predominantly lymphocytic thymomas in BUF/mna rats. Jpn J Cancer Res. 1992;83(11):1166‐1171. 1483931 10.1111/j.1349-7006.1992.tb02740.x PMC5918709

[26] Abuelhija M, Weng CC, Shetty G, Meistrich ML. Differences in radiation sensitivity of recovery of spermatogenesis between rat strains. Toxicol Sci. 2012;126(2):545‐553. 22273744 10.1093/toxsci/kfs021 PMC3307610

[27] Gruenewald DA, Naai MA, Hess DL, Matsumoto AM. The brown Norway rat as a model of male reproductive aging: evidence for both primary and secondary testicular failure. J Gerontol. 1994;49(2):B42‐B50. 8126345 10.1093/geronj/49.2.b42

[28] Syed V, Hecht NB. Selective loss of sertoli cell and germ cell function leads to a disruption in sertoli cell-germ cell communication during aging in the brown Norway rat. Biol Reprod. 2001;64(1):107‐112. 11133664 10.1095/biolreprod64.1.107

[29] Provoost AP, Van Aken M, Molenaar JC. Sequential renography and renal function in brown-Norway rats with congenital hydronephrosis. J Urol. 1991;146(2 (Pt 2)):588‐591. 1861306 10.1016/s0022-5347(17)37863-1

[30] Lahat N, Hirose W, Davies TF. Enhanced induction of thyroid cell MHC class II antigen expression in rats highly responsive to thyroglobulin. Endocrinology. 1989;124(4):1754‐1759. 2466637 10.1210/endo-124-4-1754

[31] Matsuyama M, Yamada C, Hiai H. A single dominant susceptible gene determines spontaneous development of thymoma in BUF/Mna rat. Jpn J Cancer Res. 1986;77(11):1066‐1068. 3098714

[32] Hirokawa K, Utsuyama M, Kasai M, Konno A, Kurashima C, Moriizumi E. Age-related hyperplasia of the thymus and T-cell system in the Buffalo rat. Immunological and immunohistological studies. Virchows Arch B Cell Pathol Incl Mol Pathol. 1990;59(1):38‐47. 1974097 10.1007/BF02899385

[33] Nakamura T, Matsuyama M, Kojima A, et al The effect of thymectomy on the development of nephropathy in spontaneous thymoma rats of the BUF/mna strain. Clin Exp Immunol. 1988;71(2):350‐352. 3349652 PMC1541450

[34] Noble B, Yoshida T, Rose NR, Bigazzi PE. Thyroid antibodies in spontaneous autoimmune thyroiditis in the Buffalo rat. J Immunol. 1976;117(5 Pt 1):1447‐1455. 794410

[35] Marissal-Arvy N, Mormède P, Sarrieau A. Strain differences in corticosteroid receptor efficiencies and regulation in brown Norway and fischer 344 rats. J Neuroendocrinol. 1999;11(4):267‐273. 10223280 10.1046/j.1365-2826.1999.00323.x

[36] Levy JR, Lesko J, Krieg RJ, Jr., Adler RA, Stevens W. Leptin responses to glucose infusions in obesity-prone rats. Am J Physiol Endocrinol Metab. 2000;279(5):E1088‐E1096. 11052964 10.1152/ajpendo.2000.279.5.E1088

[37] Broadhurst PL . The maudsley reactive and nonreactive strains of rats: a survey. Behav Genet. 1975;5(4):299‐319. 1191155 10.1007/BF01073201

[38] Li TK, Lumeng L. Alcohol preference and voluntary alcohol intakes of inbred rat strains and the national institutes of health heterogeneous stock of rats. Alcohol Clin Exp Res. 1984;8(5):485‐486. 6391261 10.1111/j.1530-0277.1984.tb05708.x

[39] Hansen CT, Whitney RA. Catalogue of NIH Rodents. Department of Health, Education, and Welfare 1973.

[40] Malkesman O, Maayan R, Weizman A, Weller A. Aggressive behavior and HPA axis hormones after social isolation in adult rats of two different genetic animal models for depression. Behav Brain Res. 2006;175(2):408‐414. 17069898 10.1016/j.bbr.2006.09.017

[41] Redei EE, Udell ME, Solberg-Woods LC, Chen H. The Wistar Kyoto rat: a model of depression traits. Curr Neuropharmacol. 2022;21(9):1884‐1905. 10.2174/1570159X21666221129120902 PMC10514523 36453495

[42] Rittenhouse PA, López-Rubalcava C, Stanwood GD, Lucki I. Amplified behavioral and endocrine responses to forced swim stress in the Wistar-Kyoto rat. Psychoneuroendocrinology. 2002;27(3):303‐318. 11818168 10.1016/s0306-4530(01)00052-x

[43] Snell KC . Renal disease of the rat. Pathology of Laboratory Rats and Mice. Blackwell Scientific; 1967: 105‐147.

[44] Tanase H, Yamori Y, Hansen CT, Lovenberg W. Heart size in inbred strains of rats. Part 1. Genetic determination of the development of cardiovascular enlargement in rats. Hypertension. 1982;4(6):864‐872. 6216212 10.1161/01.hyp.4.6.864

[45] Chella Krishnan K, Mehrabian M, Lusis AJ. Sex differences in metabolism and cardiometabolic disorders. Curr Opin Lipidol. 2018;29(5):404‐410. 30156571 10.1097/MOL.0000000000000536 PMC6382080

[46] Binenbaum I, Atamni HA, Fotakis G, et al Container-aided integrative QTL and RNA-seq analysis of collaborative cross mice supports distinct sex-oriented molecular modes of response in obesity. BMC Genomics. 2020;21(1):761. 33143653 10.1186/s12864-020-07173-x PMC7640698

[47] RGD . Genetic Similarity between strains in the HRDP Panel and the original HS Founder Strains. Medical College of Wisconsin. 2023. Accessed January 30, 2023. https://rgd.mcw.edu/wg/hrdp_panel/hrdp-to-hs-founder-strain-genetic-similarity/

[48] Wagner VA, Holl KL, Clark KC, et al Genetic background in the rat affects endocrine and metabolic outcomes of bisphenol F exposure. Toxicol Sci. 2023;194(1):84‐100. 37191987 10.1093/toxsci/kfad046 PMC10306406

[49] National Research Council Committee for the Update of the Guide for the C, Use of Laboratory A . The national academies collection: reports funded by national institutes of health. Guide for the Care and Use of Laboratory Animals. National Academies Press (US) Copyright © 2011, National Academy of Sciences; 2011.

[50] Grobe JL . Comprehensive assessments of energy balance in mice. Methods Mol Biol. 2017;1614:123‐146. 28500600 10.1007/978-1-4939-7030-8_10 PMC5582947

[51] Reho JJ, Nakagawa P, Mouradian GC, et al Methods for the comprehensive in vivo analysis of energy flux, fluid homeostasis, blood pressure, and ventilatory function in rodents. Front Physiol. 2022;13:855054. 35283781 10.3389/fphys.2022.855054 PMC8914175

[52] Weir J . New methods for calculating metabolic rate with special reference to protein metabolism. J Physiol. 1949;109(1-2):1‐9. 15394301 10.1113/jphysiol.1949.sp004363 PMC1392602

[53] Valerie A, Wagner KLH, Clark KC, et al Data from: Supplemental Material for: The power of the Heterogenous Stock rat founder strains in modeling metabolic disease. *Figshare*. 2023. Accessed October 30, 2023. 10.6084/m9.figshare.23632056

[54] Pfaffl M . Quantification strategies in real-time PCR. In: Bustin S (ed.), The Real-Time PCR Encyclopedia A-Z of Quantitative PCR. International university Line; 2004:87‐112.

[55] McCall MN, McMurray HR, Land H, Almudevar A. On non-detects in qPCR data. Bioinformatics. 2014;30(16):2310‐2316. 24764462 10.1093/bioinformatics/btu239 PMC4133581

[56] Sellers RS, Mortan D, Michael B, et al Society of toxicologic pathology position paper: organ weight recommendations for toxicology studies. Toxicol Pathol. 2007;35(5):751‐755. 17849358 10.1080/01926230701595300

[57] Soto JE, Burnett CML, Ten Eyck P, Abel ED, Grobe JL. Comparison of the effects of high-fat diet on energy flux in mice using two multiplexed metabolic phenotyping systems. Obesity (Silver Spring). 2019;27(5):793‐802. 30938081 10.1002/oby.22441 PMC6478533

[58] Sebo ZL, Rodeheffer MS. Testosterone metabolites differentially regulate obesogenesis and fat distribution. Mol Metab. 2021;44:101141. 33307216 10.1016/j.molmet.2020.101141 PMC7772371

[59] Wagner VA, Kwitek, AE. RGD: 401827281. Metabolic Phenotyping Data Collection. 401827281. https://rgd.mcw.edu/rgdweb/homepage/

[60] Nuttall FQ . Body mass index: obesity, bmi, and health: a critical review. Nutr Today. 2015;50(3):117‐128. 27340299 10.1097/NT.0000000000000092 PMC4890841

[61] Vantyghem M-C, Dobbelaere D, Mention K, Wemeau J-L, Saudubray J-M, Douillard C. Endocrine manifestations related to inherited metabolic diseases in adults. Orphanet J Rare Dis. 2012;7(1):11. 22284844 10.1186/1750-1172-7-11 PMC3349544

[62] Liu S, Shen S, Yan Y, et al Triiodothyronine (T3) promotes brown fat hyperplasia via thyroid hormone receptor α mediated adipocyte progenitor cell proliferation. Nat Commun. 2022;13(1):3394. 35697700 10.1038/s41467-022-31154-1 PMC9192766

[63] Mullur R, Liu YY, Brent GA. Thyroid hormone regulation of metabolism. Physiol Rev. 2014;94(2):355‐382. 24692351 10.1152/physrev.00030.2013 PMC4044302

[64] Eliason HL, Fewell JE. Thermoregulatory control during pregnancy and lactation in rats. J Appl Physiol. 1997;83(3):837‐844. 9292471 10.1152/jappl.1997.83.3.837

[65] Grases-Pintó B, Abril-Gil M, Torres-Castro P, et al Rat milk and plasma immunological profile throughout lactation. Nutrients. 2021;13(4):1257. 33920419 10.3390/nu13041257 PMC8070501

[66] Ahima RS, Prabakaran D, Flier JS. Postnatal leptin surge and regulation of circadian rhythm of leptin by feeding. Implications for energy homeostasis and neuroendocrine function. J Clin Invest. 1998;101(5):1020‐1027. 9486972 10.1172/JCI1176 PMC508653

[67] Picó C, Reis F, Egas C, Mathias P, Matafome P. Lactation as a programming window for metabolic syndrome. Eur J Clin Invest. 2021;51(5):e13482. 33350459 10.1111/eci.13482

[68] Blaak EE . Current metabolic perspective on malnutrition in obesity: towards more subgroup-based nutritional approaches? Proc Nutr Soc. 2020;79(3):331‐337. 32122428 10.1017/S0029665120000117 PMC7663313

[69] Gong DW, Murayama N, Yamazoe Y, Kato R. Hepatic triiodothyronine sulfation and its regulation by growth hormone and triiodothyronine in rats. J Biochem. 1992;112(1):112‐116. 1429499 10.1093/oxfordjournals.jbchem.a123848

[70] Gómez F, Lahmame A, de Kloet ER, Armario A. Hypothalamic-pituitary-adrenal response to chronic stress in five inbred rat strains: differential responses are mainly located at the adrenocortical level. Neuroendocrinology. 1996;63(4):327‐337. 8739888 10.1159/000126973

[71] Gómez F, De Kloet ER, Armario A. Glucocorticoid negative feedback on the HPA axis in five inbred rat strains. Am J Physiol. 1998;274(2):R420‐R427. 9486300 10.1152/ajpregu.1998.274.2.R420

[72] Whirledge S, Cidlowski JA. Glucocorticoids and reproduction: traffic control on the road to reproduction. Trends Endocrinol Metab. 2017;28(6):399‐415. 28274682 10.1016/j.tem.2017.02.005 PMC5438761

[73] Aladashvili-Chikvaidze N, Kristesashvili J, Gegechkori M. Types of reproductive disorders in underweight and overweight young females and correlations of respective hormonal changes with BMI. Iran J Reprod Med. 2015;13(3):135‐140. 26000003 PMC4426152

[74] Akana SF, Cascio CS, Shinsako J, Dallman MF. Corticosterone: narrow range required for normal body and thymus weight and ACTH. Am J Physiol. 1985;249(5 Pt 2):R527‐R532. 2998210 10.1152/ajpregu.1985.249.5.R527

[75] Iwasa K, Komai K, Takamori M. Spontaneous thymoma rat as a model for myasthenic weakness caused by anti-ryanodine receptor antibodies. Muscle Nerve. 1998;21(12):1655‐1660. 9843065 10.1002/(sici)1097-4598(199812)21:12<1655::aid-mus5>3.0.co;2-f

[76] Matsuyama M, Yamada C, Kojima A. Possible single dosage effects of the nude gene: suppression of spontaneous development of thymoma and nephropathy in BUF/Mna-rnu/+ rats. Jpn J Cancer Res. 1987;78(1):40‐44. 3102437

[77] Herold MJ, McPherson KG, Reichardt HM. Glucocorticoids in T cell apoptosis and function. Cell Mol Life Sci. 2006;63(1):60‐72. 16314919 10.1007/s00018-005-5390-y PMC2792342

[78] Ancuța E, Zamfir R, Martinescu G, Crauciuc DV, Ancuța C. The complement system, T cell response, and cytokine shift in normotensive versus pre-eclamptic and lupus pregnancy. J Clin Med. 2021;10(24):5722. 34945017 10.3390/jcm10245722 PMC8705505

[79] Lager S, Sovio U, Eddershaw E, et al Abnormal placental CD8(+) T-cell infiltration is a feature of fetal growth restriction and pre-eclampsia. J Physiol. 2020;598(23):5555‐5571. 32886802 10.1113/JP279532

[80] Klok MD, Jakobsdottir S, Drent ML. The role of leptin and ghrelin in the regulation of food intake and body weight in humans: a review. Obes Rev. 2007;8(1):21‐34. 17212793 10.1111/j.1467-789X.2006.00270.x

[81] Chabot F, Caron A, Laplante M, St-Pierre DH. Interrelationships between ghrelin, insulin and glucose homeostasis: physiological relevance. World J Diabetes. 2014;5(3):328‐341. 24936254 10.4239/wjd.v5.i3.328 PMC4058737

[82] Héliès JM, Diane A, Langlois A, et al Comparison of fat storage between fischer 344 and obesity-resistant Lou/C rats fed different diets. Obes Res. 2005;13(1):3‐10. 15761158 10.1038/oby.2005.3

[83] Druet C, Ong KK. Early childhood predictors of adult body composition. Best Pract Res Clin Endocrinol Metab. 2008;22(3):489‐502. 18538288 10.1016/j.beem.2008.02.002

[84] Santos Palacios S, Pascual-Corrales E, Galofre JC. Management of subclinical hyperthyroidism. Int J Endocrinol Metab. 2012;10(2):490‐496. 23843809 10.5812/ijem.3447 PMC3693616

[85] Green ME, Bernet V, Cheung J. Thyroid dysfunction and sleep disorders. Front Endocrinol (Lausanne). 2021;12:725829. 34504473 10.3389/fendo.2021.725829 PMC8423342

[86] Ross DS, Burch HB, Cooper DS, et al 2016 American thyroid association guidelines for diagnosis and management of hyperthyroidism and other causes of thyrotoxicosis. Thyroid. 2016;26(10):1343‐1421. 27521067 10.1089/thy.2016.0229

[87] Patel K, Joharapurkar A, Dhanesha N, et al Thyroid hormone modulates food intake and glycemia via ghrelin secretion in zucker fatty rats. Drug Res (Stuttg). 2014;64(10):523‐529. 24357139 10.1055/s-0033-1363222

[88] Nagayama Y, Nakahara M, Abiru N. Animal models of Graves’ disease and Graves’ orbitopathy. Curr Opin Endocrinol Diabetes Obes. 2015;22(5):381‐386. 26181432 10.1097/MED.0000000000000186

[89] Zhang M, Jiang W, Lu G, Wang R, Lv Z, Li D. Insight into mouse models of hyperthyroidism. Front Endocrinol (Lausanne). 2022;13:929750. 35813642 10.3389/fendo.2022.929750 PMC9257255

[90] Solberg LC, Olson SL, Turek FW, Redei E. Altered hormone levels and circadian rhythm of activity in the WKY rat, a putative animal model of depression. Am J Physiol Regul Integr Comp Physiol. 2001;281(3):R786‐R794. 11506993 10.1152/ajpregu.2001.281.3.R786

[91] Solberg Woods LC, Ahmadiyeh N, Baum A, et al Identification of genetic loci involved in diabetes using a rat model of depression. Mamm Genome. 2009;20(8):486‐497. 19697080 10.1007/s00335-009-9211-8 PMC2775460

[92] Ward AM, Syddall HE, Wood PJ, Chrousos GP, Phillips DI. Fetal programming of the hypothalamic-pituitary-adrenal (HPA) axis: low birth weight and central HPA regulation. J Clin Endocrinol Metab. 2004;89(3):1227‐1233. 15001615 10.1210/jc.2003-030978

[93] Buhl ES, Neschen S, Yonemitsu S, et al Increased hypothalamic-pituitary-adrenal axis activity and hepatic insulin resistance in low-birth-weight rats. Am J Physiol Endocrinol Metab. 2007;293(5):E1451‐E1458. 17895287 10.1152/ajpendo.00356.2007 PMC2761595

[94] Padbury JF, Do BT, Bann CM, et al DNA Methylation in former extremely low birth weight newborns: association with cardiovascular and endocrine function. Pediatr Res. 2021;91(6):1469‐1477. 33953357 10.1038/s41390-021-01531-5 PMC8568736

[95] Derakhshan A, Peeters RP, Taylor PN, et al Association of maternal thyroid function with birthweight: a systematic review and individual-participant data meta-analysis. Lancet Diabetes Endocrinol. 2020;8(6):501‐510. 32445737 10.1016/S2213-8587(20)30061-9 PMC8168324

[96] van der Spoel E, Roelfsema F, van Heemst D. Within-person variation in serum thyrotropin concentrations: main sources, potential underlying biological mechanisms, and clinical implications. Front Endocrinol (Lausanne). 2021;12:619568. 33716972 10.3389/fendo.2021.619568 PMC7945716

